# A new neonatal BCG vaccination pathway in England: a mixed methods evaluation of its implementation

**DOI:** 10.1186/s12889-024-18586-8

**Published:** 2024-04-26

**Authors:** Koren Jones, Georgia Chisnall, Tim Crocker-Buque, David Elliman, Jeremy Horwood, Sandra Mounier-Jack, Colin NJ Campbell, Vanessa Saliba, Tracey Chantler

**Affiliations:** 1 UK Health Security Agency, London, England; 2 London School of Hygiene and Tropical Medicine, London, England; 3 Great Ormond Street Hospital, London, England; 4 University of Bristol, Bristol, England

**Keywords:** Evaluation, Tuberculosis, Bacillus calmette-guerin, Vaccine, England

## Abstract

**Introduction:**

The introduction of a national evaluation of newborn screening for Severe Combined Immunodeficiency (SCID) in England triggered a change to the selective Bacillus Calmette-Guerin (BCG) vaccination programme delivery pathway, as this live attenuated vaccine is contraindicated in infants with SCID. The neonatal BCG vaccination programme is a targeted programme for infants at increased risk of tuberculosis and used to be offered shortly after birth. Since September 2021 the BCG vaccine is given to eligible infants within 28 days of birth, when the SCID screening outcome is available. We explore the experiences of those implementing the new pathway, and how they made sense of, engaged with, and appraised the change.

**Methods:**

A mixed-methods evaluation was conducted between October 2022 and February 2023. This involved national online surveys with BCG commissioners and providers and qualitative semi-structured interviews with commissioners, providers, and Child Health Information System stakeholders in two urban areas. Survey data was analysed using descriptive statistics and interview data was analysed thematically. The data was triangulated using Normalization Process Theory as a guiding framework.

**Results:**

Survey respondents (*n* = 65) and qualitative interviewees (*n* = 16) revealed that making sense of the new pathway was an iterative process. Some expressed a desire for more direction on how to implement the new pathway. The perceived value of the change varied from positive, ambivalent, to concerned. Some felt well-prepared and that improvements to data capture, eligibility screening, and accountably brought by the change were valuable. Others were concerned about the feasibility of the 28-day target, reductions in vaccination coverage, increased resource burden, and the outcome of the SCID evaluation. New collaborations and communities of practice were required to facilitate the change. Three main challenges in implementing the pathway and meeting the 28-day vaccination target were identified: appointment non-attendance; appointment and data systems; and staffing and resourcing. Feedback mechanisms were informal and took place in tandem with implementation.

**Conclusion:**

The new NHS neonatal BCG service specification has created an effective structure for monitoring and managing the BCG vaccination programme, but further work is required to support delivery of the 28-day vaccination target and improve uptake rates.

**Supplementary Information:**

The online version contains supplementary material available at 10.1186/s12889-024-18586-8.

## Introduction

Tuberculosis (TB) is an infectious disease caused by the Mycobacterium tuberculosis complex bacteria and transmitted by the respiratory route [1]. It is a serious infection that primarily affects the lungs but can also affect other organs [2]. TB is the second leading cause of infectious disease mortality globally after COVID-19, despite it being curable and preventable [2]. In 2021, 4,425 people were notified with TB in England and rates remain highest in people born outside of the UK, with social risk factors e.g., homelessness, and from deprived communities [1]. The UK Health Security Agency (UKHSA) and NHS England (NHSE) are committed to meeting the World Health Organisation TB elimination targets through their TB action plan [3].

The Bacillus Calmette-Guerin (BCG) vaccine was developed in 1921 and remains a vital component of the preventative strategy against TB, alongside intensive treatment and contract tracing. In England, the BCG vaccination programme was introduced in 1953, and was initially offered to 14-year-old adolescents. By the 1960s, the TB burden had shifted to new migrants from high-prevalence countries. Recommendations were subsequently made to add a selective neonatal BCG vaccination programme, targeted at individuals based on TB exposure risk. From 2005, the adolescent BCG programme was stopped [4]. The primary component is now the neonatal programme, although BCG is also offered to older children and adults if required [5]. The neonatal BCG immunisation programme aims to protect at-risk infants from the more serious childhood forms of TB [4] and infants eligible for neonatal BCG vaccination include [6]:

### BCG neonatal eligibility

- All infants (aged 0 to 12 months) whose parent/s or grandparent/s were born in a country where the annual incidence of TB is 40/100,000 or greater.
- All infants (aged 0 to 12 months) living in areas of England where the annual incidence of TB is 40/100,000 or greater.

In September 2021, the BCG vaccination programme underwent further reform in response to an evaluation of the addition of Severe Combined Immunodeficiency (SCID) to the newborn blood spot test conducted at 5 days of age, as recommended by the UK National Screening Committee (UKNSC) [7]. SCID is a rare, inherited condition which results in severely impaired immune system functioning and death within infancy due to infections, if untreated [8]. It is estimated that around 15 to 25 infants are born with SCID each year in the UK, with an incidence rate of around 1 in 40,000 [9]. The Joint Committee on Vaccination and Immunisation (JCVI) recognised the dilemma introducing SCID screening had for the timing of BCG vaccination [7]. Because BCG is a live attenuated vaccine, it is contraindicated in infants with SCID due to the elevated risk of serious complications, such as disseminated BCG disease (BCGosis) and death [10]. Hence, the JCVI recommended that BCG vaccination should be moved to 28-days of age, at which point SCID screening results would be available, preventing vaccination of infants with SCID [6].

This change was implemented in September 2021, and monitoring BCG vaccination uptake among eligible infants at 28 days of age (with a target uptake of 80%) was included as a key aim of England’s TB action plan (2021 to 2026) [3]. SCID screening was introduced in six evaluation areas (Manchester, Birmingham, Sheffield, Newcastle, London Great Ormond Street Hospital and London Southeast Thames), representing two thirds of infants born in England. To ensure consistency and safety for all infants in England the neonatal BCG vaccination programme was revised nationally, with a holistic shift in delivery of BCG from birth to day 28 (and checking for a SCID screening result) irrespective of whether infants live within SCID evaluation areas.

**Fig. 1:**
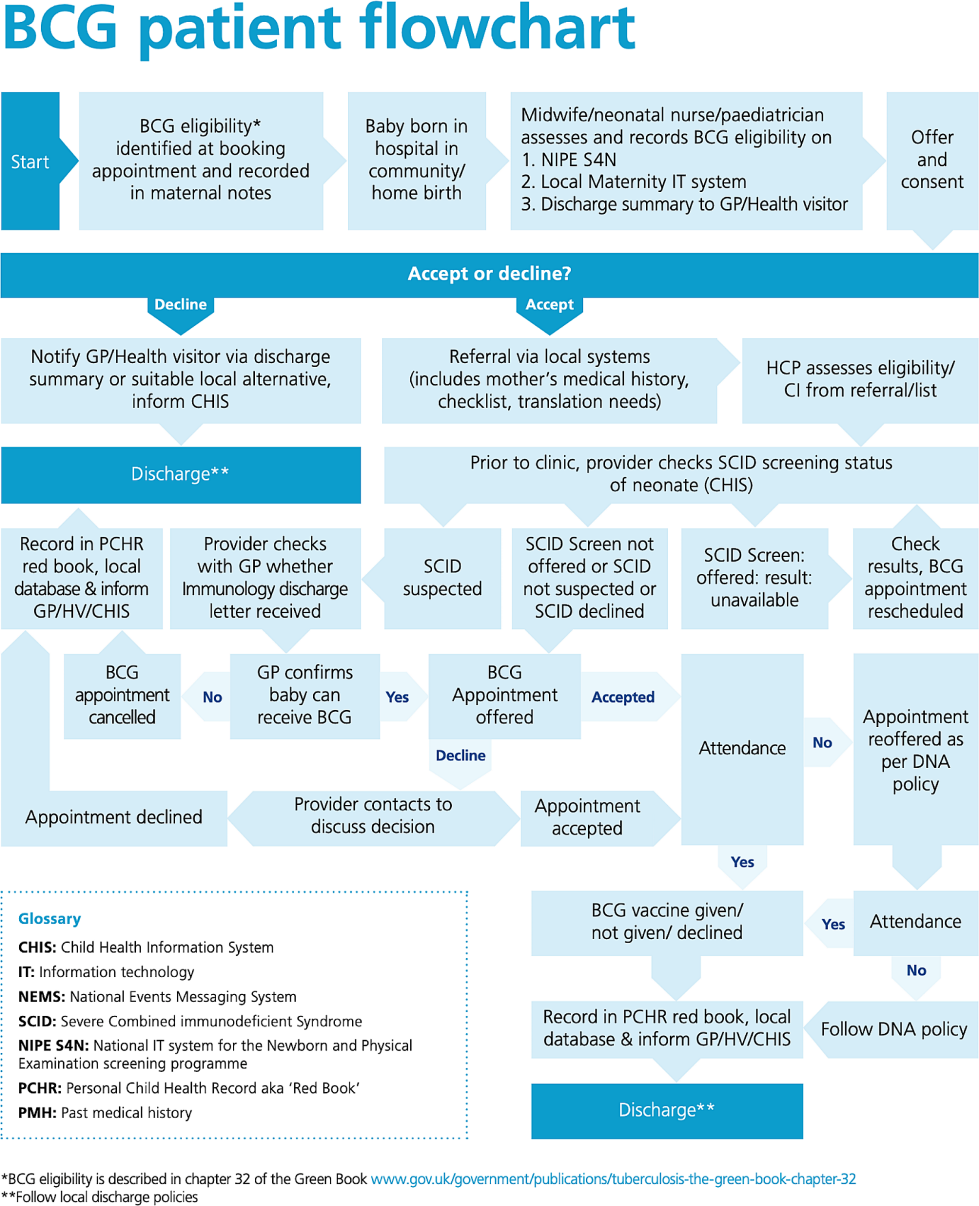
BCG Patient Flowchart [11] *Note* From UK Health Security Agency (2021) [11]. Crown copyright 2021. Reprinted with permission

The decision to move the BCG vaccine offer from birth to day 28 triggered major changes in the commissioning and operational delivery of the NHS neonatal BCG programme. A revised national service specification (S7A) and a new vaccination patient pathway and information resources for health professionals and the public were developed (Fig. 1) [11]. Prior to the change BCG was commonly delivered in maternity units prior to discharge [12]. BCG is now delivered after a negative SCID result in evaluation areas where the screening is offered or following a SCID screening not offered result in non-evaluation areas, via an outpatient model in the community ([7, 12]).

Concerns were raised that delaying the BCG vaccination could result in more neonatal TB infections and lower BCG vaccine uptake, disproportionately affecting deprived and ethnically diverse populations ([12, 13]). Pillay et al. (2022) argued that this risk must be limited by effective, sustained, and cohesive working between different NHS sectors. Common barriers to vaccine uptake and strategies to reduce health inequalities were flagged, including language barriers and the need for multilingual, targeted, multi-agency engagement. Other pre-empted challenges included health beliefs regarding proximity to other vaccines in the routine schedule and deferral of vaccination due to mild illness [13].

The implications of offering both SCID screening and BCG vaccination is an interesting global phenomenon, without a clear consensus. The largest SCID screening programme is in the USA, where the BCG vaccination is not widely used [14]. In countries offering both, the approach has varied significantly. For example, Norway and Taiwan recommend BCG vaccination at six-weeks old and five to eight months old respectively, while Australia and New Zealand have SCID screening and recommend neonatal BCG vaccination without waiting for SCID results [14]. The results of the SCID evaluation will determine whether SCID screening will become routinely delivered within England [13]. The UKNSC, together with UKHSA, will consider the impact on the BCG vaccination programme, TB incidence, BCGosis, and the experiences of commissioners and providers. BCG vaccination coverage at 3 months of age increased consistently in England between quarter 1 2022/2023 and quarter 4 2022/2023, (the period where BCG coverage data is available), from 63.4 to 70.4% (+ 11.0%). Notably, the 80% coverage target has not been met in any quarter, despite coverage being recorded at 3 months instead of 28-days of age.

This evaluation seeks to explore commissioners and providers’ experiences of implementing the new pathway, and how they made sense of, engaged with, and appraised the change. This includes the identification of implementation challenges, examples of good practice, and areas for consideration going forward as commissioners and providers strive to increase coverage and meet the 28-day uptake target.

## Methods

### Evaluation design

This evaluation combined qualitative and quantitative data, in a mixed methods evaluation design to investigate current practice and capture the experience and perspectives of service commissioners and providers (see Table 1). This consisted of qualitative interviews (October 2022-February 2023) in two urban areas where the SCID evaluation was taking place and national questionnaire surveys (November 2022). Initially, the two workstreams were going to produce separate outputs, but once the data was available it was decided that most value could be gleaned from reporting the data in tandem. This enables the evaluation to provide a national overview and rich explanatory accounts.

Survey and qualitative data were analysed separately and subsequently synthesized by applying Normalization Process Theory (NPT) as a guiding framework [15]. NPT is an established theory for exploring the implementation of complex interventions and the integration of new interventions into routine practice. It explores how a change is understood (coherence), how a community of actors coalesce around the change (cognitive participation), put into practice (collective action), and appraised it (reflective monitoring). Strengths and limitations of this evaluation are considered in the discussion.

**Table 1:** Targeted evaluation participants

Role of participant	Summary of responsibilities
Public Health Commissioners	Public health commissioners fund, plan, agree and monitor services to improve the health and wellbeing of their population e.g., the BCG vaccination programme [16].
Providers	A health care provider is an organisation acting as a direct provider of public health services [17]. Experiences of various BCG vaccination service providers were captured in this study, including NHS trusts, community trusts, maternity services, and specialist TB services.
Child Health Information System (CHIS)	A CHIS is an NHS commissioned service that is responsible for collating data from various organisations for all children aged 0–19 that are either residents or registered with a GP practice in a specified area, into a single Child Health Record [18]. Data is received from various organisations to help with increasing vaccination coverage, supporting the healthy child programme and assisting in the delivery of children’s public health services [18].

### Qualitative methods

#### Sampling and recruitment

We selected two urban areas that were part of the SCID screening evaluation for the qualitative component of this study. This means that SCID screening was conducted in these areas and results would be available prior to BCG vaccination. Due to the limited number of people overseeing this change in each area, the interview areas are redacted to uphold the confidentiality of our interview participants. Capturing experience of implementation is critical to understanding what worked well and where modifications relating to aspects of the pathway were required. Site selection was informed by geography, the prevalence of TB in these areas and what types of health organisations (e.g., maternity units, community clinics, CCGs/primary care) were involved in delivering the BCG vaccination programme. Our study sites included commissioners and providers that had to make larger or smaller changes in terms of service delivery to introduce the new BCG pathway.

In each area the study team mapped the provision of BCG vaccination services with the support of commissioners and sent email invites the commissioners and all providers to voluntarily participate in an interview. Potential participants received a study information letter and were given the opportunity to ask questions about the evaluation before deciding to take part. Written informed consent was given by all participants prior to the interviews, which were conducted by LSHTM researchers (TC, TC-B). This workstream was granted ethical approval by the UKHSA Research Support and Governance Office (Ref: NR0328).

#### Data collection

Interviews followed a topic guide to ensure that interviews covered similar themes, but there was flexibility to ensure participants could talk about things they felt were important. Copies of the topic guides can be found in the additional pdf files supplied [see Additional file 1 and 2]. The interview topic guide was developed using NPT as a guiding theoretical framework. All interviews were conducted virtually (using Microsoft Teams or ZOOM) and were audio recorded; some were transcribed using an automated transcription function (Otter.ai) and some were transcribed by a company that signed a confidentiality agreement. Transcripts were reviewed and cleaned in conjunction with audio recordings. Interview data was collected on encrypted, and password protected audio-devices and computers and stored (in compliance with the 2018 Data Protection Act) in a secure LSHTM data storage folder, that only LSHTM researchers (TC, TC-B, GC) could access via a double authentication process.

#### Data analysis

Interview transcripts were analysed using the framework method, a form of thematic analysis [19]. Framework analysis provides a systematic and comprehensive method of drawing conclusions from qualitative data [20]. Framework analysis involves seven core stages [19]: transcription, familiarization, coding, analytical framework development, application of the analytical framework to the transcripts, charting data into a framework matrix, and interpretation. Codes were built into the analytical framework using NPT theory as a guiding holistic framework (coherence, cognitive participation, collective action, and reflexive monitoring) (GC, TC). While a theoretical framework was used, themes within different components of the framework emerged from the data, hence, the analysis used a blended deductive-inductive approach. The analytical framework was built into NVIVO 12 (a qualitative analysis software produced by Lumivero) and applied to the transcripts (GC, TC) with some segments of text assigned to multiple codes.

### Quantitative methods

#### Sampling and recruitment

The sample for the quantitative component of this study included all NHSE regions (East of England, London, Midlands, North East and Yorkshire, North West, South East, South West), including SCID evaluation and non-SCID evaluation areas. Non-SCID evaluation areas are still required to check for SCID screening results prior to issuing BCG vaccination, even though (unless a family have moved within the baby’s first few weeks of life) it may simply be recorded as ‘SCID screening not offered’.

This workstream was also granted ethical approval by the UKHSA Research Support and Governance Office (Ref: NR0328). A cover page informed participants of the purpose of the evaluation, that their answers would be confidential, and that the data would be stored in line with the Data Protection Act 2018. No special category data (e.g. race or ethnic origin) was collected in this questionnaire survey. As an online survey, implied consent was deemed appropriate, whereby participants opted into the survey by deciding to complete and submit the form.

#### Questionnaire design and dissemination

Two online questionnaires were developed to capture experiences of the change and implications for practice nationally (KJ, VS). Copies of the online questionnaires can be found in the additional pdf files supplied [see Additional file 3 and 4]. The questionnaires were designed using SelectSurvey (an online survey software); one for regional BCG commissioners and one for BCG providers and CHIS. Prior to rollout the survey was piloted with Screening and Immunisation colleagues (NHS England). Links to both questionnaires were shared via the NHSE Public Health Commissioning bulletin on the 3rd and 24th of November 2022. NHSE regional public health commissioning leads or nominated deputies were invited to complete the commissioner questionnaire on behalf of their region and to circulate the provider questionnaire link to all BCG vaccination programme providers in their region. Potential respondents were given 4 weeks to complete the questionnaires.

#### Data analysis

The questionnaire data were extracted from SelectSurvey on 7th December 2022 for analysis. This data was stored securely in a restricted folder that could only be accessed by UKHSA researchers (KJ, CC, VS) directly involved in the quantitative data analysis. The rating, multiple choice and closed-ended questions were analysed using descriptive statistics. Analysis of the free-text responses was conducted alongside the qualitative data analysis using a form of thematic analysis, as outlined above. Case studies were highlighted, describing examples of good practice.

## Results

### Evaluation participants

There were 11 commissioner and 54 provider questionnaire survey respondents, with responses to both surveys received from all England NHS regions. Responses were received from providers covering 93/153 local authorities in England, including both SCID and non-SCID evaluation areas. Most providers (96%) had been commissioned to deliver the BCG vaccination programme prior to the change implemented in September 2021. We conducted 11 semi-structured interviews with 16 interview participants from the 2 urban study areas. Interview participants included 8 providers, 5 commissioners and 3 CHIS managers. Data was triangulated across both data sources, the full survey reports for commissioners and providers can be found in Additional files 5 and 6.

Commissioner roles were most often described by participants as ‘Screening and Immunisation Manager’, other variations included ‘Screening and Immunisation Lead’ or ‘Screening and Immunisation Coordinator’. Providers across the implementation chain were represented, from immunisation nurses through to clinic managers. Provider roles included ‘BCG Immunisation Nurse’ or ‘TB Specialist Nurse’ through to ‘BCG Immunisation Team Lead’, ‘BCG Coordinator’, and ‘Immunisation Service Manager’. CHIS referred to themselves as ‘CHIS manager’ or ‘CHIS project coordinator’.

### Coherence (making sense of the change)

Making sense of the new pathway was widely reported as an iterative or reactive process. This sentiment was identified in both the qualitative and quantitative workstreams. In part, this may be attributable to the fact that the BCG pathway was introduced in September 2021 during the COVID-19 pandemic, with 64% of commissioners reporting that this impacted their ability to prepare for the change. Beyond the pandemic, the process was iterative due to trialing different ways of putting the pathway into practice and the emerging scale of the change:

…the change to accommodate SCID screening sounds in theory like a very straightforward process, but actually very quickly we understood that it had massive implications right the way through from midwives, screening link midwives, CHIS, the lab, BCG providers, primary care going forward, the patients, the families. It was enormous…(Commissioner)

…*a lot of learning on the job, a lot of going back and sitting back, going oh, we haven’t thought about that…*(Commissioner)

There was a wide range of experiences when it came to deciding how to implement the new pathway across all participant groups (providers, commissioners, and CHIS), some felt well supported, whilst many expressed the need for more direction on how to implement centralised guidance locally. Survey respondents cited late publication of guidance and a short implementation period as contributing factors.

*Well, it was relatively quite straightforward, really, which is unusual for us because it’s usually lastminute.com with programme changes.* (CHIS)

...*fraught few months to be honest with you.* (CHIS)

*And there was a lot of awful lot of stuff that we were looking for guidance for and they were very much saying that’s a local decision you need to decide which was really difficult when it was like containing sand at one point it was so difficult. Anyway, we did it so that was fantastic. That was a great outcome.* (Commissioner)

…*a bit more directional information, but that never came basically. And you just have to get on with it, you know, do what you can do*. (Provider)

Some felt that the diffusion of responsibility to local regions, while well intended, made the pathway implementation challenging. Some reported that more applied guidance and clearer designation of responsibility would have made the implementation process easier and less stressful. This was particularly true given the scale of the change being implemented and its far-reaching impacts across the delivery pathway. Both interview and survey respondents cited that the provision of national BCG training would be welcomed.

From interview data, it could be deduced that people’s relationship with the iterative nature of the sense-making process seemed to depend on whether they felt the issue should have been foreseen or whether such occurrences are unavoidable: “*Which was not necessarily something that anybody could have anticipated. And we did try to anticipate most things and I thought it was relatively seamless. I think I can say with some confidence now, but at the time we didn’t feel it was just a bit trepidatious…sometimes you don’t realize where it’s going wrong till it’s gone wrong and then you can fix it." *(Commissioner).

The extent to which people perceived the benefits and importance of the change ranged from positive, ambivalent (or neutral), through to concerned. Those who were positive about the BCG pathway change felt that they knew about the change for a long time: “*We were pre-warned we were expecting it.*” (Provider). Improved data capture, accountability, and more robust eligibility screening for the BCG programme was consistently reported as the primary benefit in both data workstreams, particularly as BCG eligibility and coverage is now captured as part of the national COVER dataset: “*I think there being a bigger spotlight on it now probably means that it’s more robust in terms of delivery. So maybe we’ve got more accountability for the babies now…when I first qualified as a midwife, I worked somewhere that we had a universal offer, so we used to catch all our babies on the way out of the wards, but if you missed them, they were gone."* (Commissioner).

Some commissioners reported that staff were ambivalent towards the new pathway, and that this posed a challenge in enlisting support for the programme change. Conversely, strong leadership from commissioners and good engagement from providers was cited as a defining feature in successfully implementing the new programme. Those who were concerned about the new pathway consistently discussed one of four things, all of which are supported by both data workstreams. Firstly, the feasibility of the 28-day target (and its rationale):

*I personally feel the 28 days is not necessarily achievable to level of of 80% which is being asked. I think 3 months or 2 months would be a better target.* (Commissioner)

…*I understand why obviously it has gone from being delivered pretty much straight after birth, to how can we fit SCID in and still give it as early as possible…although there is a national task and finish group for BCG and one of the other regions asked for a rationale on the 28-day target and we are still waiting for that to come back*… (Commissioner)

Secondly, increased Did Not Attend (DNA) rates. Protecting vulnerable infants through SCID screening was not frequently mentioned as a benefit of the BCG programme change, despite it being the reason for the change. There was concern about the impact of the programme on vaccination coverage and in a minority of interviews there was explicit skepticism over whether the trade-off between SCID screening and increased DNA was a worthwhile trade-off: “*Honestly. Go back, go back to, go back to a universal offer. But in maternity unit. I think you’ll pick more babies up."* (Commissioner).

Thirdly, resource pressures. When asked about the impact of the new pathway, many referred to the additional strain placed on staffing and resources. Many felt that this was an unintended consequence which had not been considered during the conceptualisation of the pathway change: “*And I think it is fair to say and I know the national team have commented this also that that the change to accommodate SCID screening sounds in theory like a very straightforward process, but actually very quickly we understood that it had massive implications and change right the way through from midwives, screening link midwives, CHIS, the lab, BCG providers, primary care going forward, the patients, the families. It was enormous…"* (Commissioner).

Fourthly, the changes being driven by the SCID evaluation with no guarantee that the changes would be permanent: “*It’s still a pilot. So, if and where obviously a pilot area, but if they come back nationally and say. Actually, if the pilot of SCID not works and you need to go back to a previous model. What happens then*?” (Commissioner). This was particularly poignant for those who were also not part of the SCID pilot.

### Cognitive participation (creating a community of practice)

New communities of practice were needed to implement the pathway change. This required new collaborations between vaccination and screening teams due to the newfound interdependence of their programmes. The value of these new working relationships and communication channels were identified by both workstreams. There is no other vaccination programme which is interdependent on another programme (i.e., screening) run by different teams that requires the timely publication and access to dataflows in real time.

Initially, it took some encouragement to enroll people into this new community of practice and articulate the need for them to be involved: *“I think the worst we ever experienced from any of our providers was more kind of a lack of engagement rather than any resistance or reluctance…and probably not quite fully understanding how that would impact them particularly…perhaps providers that we had to spend a little bit more time with to encourage them to be part of that full effort…we probably started those multidisciplinary stakeholder meeting around May and I think we continued them through until about March, April of this year."* (Commissioner).

This new community of practice was credited as a positive outcome of the change, with all actors involved gaining greater understanding and appreciation of the roles played by one another: “…*a positive that came out of this was that stakeholders who previously maybe worked quite in isolation, having everybody together in one place, it does give you appreciation of how important everybody’s roles are and particularly CHIS I think that previously the importance of child health holding all information in one place maybe had been underestimated, whereas it became apparent that they were linchpin. Really, that the whole thing would fall apart if it wasn’t for CHIS."* (Commissioner).

### Collective action (putting the change into practice)

Several challenges and solutions were identified with the implementation and delivery of the new BCG vaccination pathway which are outlined below. Identified challenges and solutions were consistent across the interview and survey workstreams. Commissioners rated the implementation of the change on a five-point scale between ‘poor’ and ‘excellent’. The ratings were good (*n* = 5, 45%), neutral (*n* = 3, 27%) and fair (*n* = 3, 27%), with no commissioners rating the implementation either poor or excellent. All commissioners (100%, *n* = 11) reported implementation challenges, whilst 73% (*n* = 7) noted implementation benefits.

Some commissioners reported that the COVID-19 pandemic impacted implementation (45%) and programme delivery (45%) due to staff illness, clinic cancellations and families cancelling BCG vaccination appointments due to illness or COVID-19 anxiety. Commissioners noted that this could have resulted in lower vaccination uptake, delays to vaccination, and the creation or increase of backlogs. Many felt that backlogs associated with the time taken to imbed the new system were posing a challenge in meeting current targets:

*But for the future, our future planning for neonatal BCG, our ambition is to get 80% uptake or more up four weeks. So, we’ve got work to do around backlogs and management of clinics.* (Commissioner)

…*there was a backlog in [site name redacted] of over 450 babies.*.. (Provider)

There was consensus in the survey and interview responses that meeting the 28-day vaccination target was the primary challenge in delivering the new BCG vaccination pathway. Commissioners were asked to quantify how many BCG vaccinations had been delivered at or before 28-days in the first ten months of the new pathway (01/09/2021–30/06/2022). Low adherence to the target was reported, with 20% (*n* = 2) reporting < 20%, 30% (*n* = 3) reporting between 20% and 39%, and 50% (*n* = 5) reporting between 40% and 59%, whilst one commissioner did not provide a response. Three key challenges in implementing the new pathway and meeting the 28-day target were identified which are outlined below.

### Vaccination uptake

DNA rates posed a significant challenge in meeting the 28-day target for most providers. The appointment DNA rates reported by providers varied, with 48% reporting DNA rates of less than 20%, whilst 52% reported DNA rates between 20% and 59%. Many felt that this challenge had been “*underestimated*” (Commissioner) and that this was inherently (but not exclusively) associated with moving from a bedside to a community-based model of delivery: “…*it’s easier with a captive audience, isn’t it? If they’re already in hospital you give them the vaccine and then they go home, but it’s trying to get them to come back again at a later date…"* (CHIS).

Many were unclear about the reasons for DNAs and how to best address meeting the 28-day target. When asked how DNA rates could be improved responses included “*not sure*” (provider) and “*no idea*” (Provider). Many referred hesitantly to parental decision-making and cultural norms but acknowledged that active refusal was “*…few and far between*” (Provider). There was a shared understanding that there is “*still work to be done between [CHIS] and providers to understand why the figures don’t look better."* (CHIS).

Others drew on their individual experiences of delivering the BCG programme to share potential reasons for DNA’s. Challenges such as English literacy and demographic related health inequalities were reported in both data workstreams, although they acknowledged that these affect all vaccine programmes and not just BCG. Approximately half of provider survey respondents who gave details on their provision of additional information (52%, *n* = 16) reported that beyond the translation of centrally provided flyers no bespoke language support was offered. 74% Providers felt that improved vaccine education and literacy around the BCG programme was needed, particularly considering the programme change: “*Well actually, did they not inform you we actually have to wait for your SCID results. Oh, SCID results. What’s that? And we have to have that conversation."* (Provider).

Providers who were interviewed reported that parents were often concerned by the proximity of the BCG vaccination to the 8-week vaccination schedule and were surprised that the vaccine used to be given at birth. When given the choice parents may defer vaccination until a child is a bit older: “…*we do try to explain that it was given on the day of birth before in the maternity wards, which parents are always shocked by, but if they’ve got a choice in the matter, which indeed they do, then they would rather wait.*” (Provider). Similarly, survey respondents suggested that parents may want more time to consider the vaccination.

Clinic accessibility was another challenge reported in the interviews and survey responses that may be contributing to DNA rates. At the time of survey completion (November 2022), 44% of providers (*n* = 24) reported that they had not completed an accessibility assessment. Daytime clinics were the most common, with 76% of providers (*n* = 41) offering daytime only clinics, with no weekend or evening clinic provision. Travel distance was also highlighted, with 57% of providers (*n* = 31) reporting a maximum travel distance of at least 10 miles, which was particularly challenging for families reliant on public transport.

There were several examples of good practice from both workstreams to overcome this challenge, including ensuring that BCG vaccination clinic timings were convenient for families, heat mapping where families live to select clinic locations, and offering parents flexibility in their choice of clinic location and appointment timings. Other examples of good practice included: phoning parents and ensuring allocated appointments were acceptable, rescheduling unsuitable appointments prior to DNA occurring, and sending text reminders in the primary language of the recipient. One CHIS provider expressed interest in alerting GPs to unvaccinated infants who could be signposted back to the BCG service.

### Appointments and data systems

Developing appointment and data systems to meet the 28-day target was challenging. Implementing the new BCG vaccination pathway required additional eligibility screening and referral processes, involving multiple stakeholders, which took time to embed. Setting up data systems between CHIS and providers was initially challenging with delays in receiving results or BCG eligibility not being completed on the computer system (S4N). Some providers noted that systems had been developed to overcome this: “*I say it was difficult trying to get the SCID result in setup, but we’ve conquered that now.*” (Provider). Survey respondents felt there was a need to improve recording of BCG eligibility recording on S4N, suggestions included making it a mandatory field or revising the national template to make it easier to complete.

Some providers reported that when SCID results had not been shared by CHIS providers they were able to use alternative data systems to access the results directly, although this caused additional administrative burden. Both workstreams identified that the data systems struggle to monitor infants who have moved in/out of area, and this is an ongoing area which is being addressed: “*I still think we’ve got some work to do around the movement in pathway.*” (CHIS). One example of good practice was GPs and health visitors acting as a failsafe for infants who were not referred for BCG or those who had three DNAs and had been discharged. The survey workstream also suggested that the new pathway had promoted better knowledge regarding the programme and BCG eligibility, but that there were still issues with eligible infants not being referred or inappropriate referrals.

The small window available to book in and vaccinate infants between the availability of the SCID result and the 28-day target was considered challenging by some providers: “…*to try and meet that 28-day but currently it’s really difficult because if we have to wait for the SCID result, that’s 21-days. It doesn’t give us enough time to book an appointment well in advance…*” (Provider). Although, survey data reported that most providers (74%) were booking the vaccination appointment at referral, ahead of the SCID result becoming available. Providers who used this appointment booking system reported that it was a suitable approach, and that “*nine times out of ten*” (Provider) the results would be available ahead of the scheduled appointment.

### Staffing and resourcing

Staffing and resourcing were another challenge in meeting the 28-day target. Many felt this was an unintended consequence not fully considered during conceptualization of the pathway change. This was widely attributed to the changes to appointment and data systems, which resulted in a sizable administration burden including searching and screening SCID results, appointment booking, reminders, recall and data input: “…*making sure the letters and the paperwork and are getting done every week. And as I say, whereas I didn’t have to do that before…It’s a lot more work involved now than what it was.*” (Provider). The additional administrative burden often fell on BCG vaccinators, and many services were already short staffed: “Y*ou’re the band 6. All you should be doing is vaccinating the baby. And you think that doesn’t work with that, you know, because there’s a lot of work involved in it.”(Provider).* However, this experience varied between providers, exemplified by the number of BCG vaccine appointment reminders; 22% sent no reminders, whilst 33% sent one reminder, 30% sent two reminders, and 15% sent three or more reminders.

Consequently, many programmes have built capacity across several roles (e.g., administrative staff, coordinators, and vaccinators) to deliver the new pathway. Those who have been able to expand capacity cited how this was fundamental in delivering the pathway. Expanding the capacity for BCG vaccinators is particularly challenging given the shortage of qualified staff and the additional training requirements needed to deliver BCG: “…*to get a BCG nurse trained and up to speed and have to deliver this program you’re looking at three months minimum. It’s just so difficult to find trained nurses to deliver this program and to the level it needs to be delivered at.*” (Commissioner).

Beyond staffing, other resource constraints included securing an appropriate clinic location and funding: “Y*eah, and funding. I think trying to deliver a BCG program on potentially £15.00 for an urgent service is not even close to the bar really, and there needs to be some specific amount of funding to deliver these programs*.” (Commissioner). Examples of good practice included the introduction of a new contract in one region which facilitated ‘blocked’ rather than ‘per item’ payment and building sustainable implementation pathways using a combination of staff groups.

### Reflective monitoring

From the start, the implementation of the new BCG pathway was an integrative and reflexive process as reported within the coherence building section. Consequently, the process of reflexive monitoring was very much embedded within, and acting in tandem to, the sense-making and implementation process whereby several refinements were made early on (e.g., how SCID results were accessed, when BCG vaccination appointments were being booked). These appraisal and feedback mechanisms were widely informal taking the form of candid conversations and team meetings among the devised communities of practice. None of the commissioners who undertook the survey reported conducting evaluations of the case studies they presented.

*Consulting with every stakeholder at that point so that they could raise what progress they’ve made, what challenges they’ve got and that we could try and work our way through it…* (Commissioner)

*We were meeting with everybody who was involved in this. So, the lab and CHIS and maternity and you know, BCG providers really regularly and trying to just keep up to date with making sure that everybody receives the information as it as it was coming through…* (Commissioner)

## Discussion

This study explored commissioners, CHIS, and providers’ experiences of implementing the recent change to the BCG vaccination pathway. Through using NPT, we were able to explore how staff made sense of the change, formed a community of practice, delivered the operational work needed to implement the new pathway (with a focus on implementation challenges and examples of good practice), and understood the appraisal work going into reconfiguring implementation. Here we re-visit the central findings and compare them with the potential challenges and opportunities raised within opinion pieces published ahead of the change. Where appropriate, we also situate our findings within wider literature. At the end of the discussion, we present key areas of consideration for policy stemming from the findings of this study.

Contextually, it is important to note that this change was implemented during the COVID-19 pandemic (September 2021) which impacted commissioners’ ability to prepare for and implement the change alongside ongoing service delivery. This was predominantly due to staff and patient illness, COVID-19 anxiety, and appointment cancellations, all of which have been reported as wider barriers to healthcare delivery during the COVID-19 pandemic [21]. The challenge of implementing a complex programme change at a time of constrained resource had implications for BCG vaccination uptake and timeliness of vaccination, a phenomenon which has been reflected in other childhood vaccination programmes globally [22]. BCG vaccination backlogs created while the new program was implemented (whether this be due to COVID-19 disruption or time taken to bed in the new system) continue to drain staff resources and skew performance ratings, despite significant improvements in the implementation of the new pathway. It is difficult to fairly assess the performance of the BCG vaccination service until these backlogs have been cleared and resources are fully allocated to providing a prospective service. While it is important to acknowledge the contextual challenges posed by the COVID-19 pandemic and service backlogs this does not diminish the wider challenges identified with the implementation of the new pathway, or the opportunity posed for programme learning and adaption going forward.

This evaluation identified three core challenges in meeting the 28-day target: DNA rates; data and appointment systems; and staffing and resourcing. DNA was consistently reported as one of the most significant challenges in meeting the 28-day target and was a concern raised within several publications ahead of the programme change ( [12, 13]). Academic and NHS Trust colleagues voiced a need to proactively cater for general barriers to vaccination such as language barriers and advocated for the use of tailored, multi-agency interventions [13]. They also noted the risk of negative health beliefs regarding the proximity of BCG vaccination to other vaccines given as part of the routine programme at 8-weeks of age.

Despite these concerns, commissioners and providers within this study voiced that the challenge of DNA had been “underestimated,” and the loss of a captive audience was having implications on vaccination uptake. There was a shared understanding that more work was needed to better understand and respond to poor vaccine uptake, although many felt active vaccine refusal was unlikely to be the primary reason; reflecting the findings of several studies which have found refusal of childhood vaccinations to be uncommon within the UK ( [23–26]). Instead, there is growing need to better understand DNA as a separate phenomenon to vaccine refusal [23].

Pillay et al.’s concern regarding health education and the need to address the proximity of other routine vaccinations [13] was also a finding of this study, however further communication regarding SCID, and the recent programme change were recommended. Study participants shared the view of Pillay et al. that English literacy and other health inequalities were likely barriers [13]. Given this, it is concerning that at the point of data collection most providers offered limited appointment times, clinic locations, and limited additional language support. Furthermore, 44% of providers reported that they had not yet completed their accessibility assessment.

We recommend that all providers complete outstanding accessibility assessments and implement additional, tailored provision where necessary. Due to the selective nature of BCG, the impact of this pathway change disproportionately falls on those from deprived and ethnically diverse populations [12]. Hence, it is particularly important that service accessibility is addressed. Vaccine service-related provision (i.e., number of reminder letters, appointment availability, provision of educational material, etc.) varies considerably within the routine vaccine programme [23]. It is essential that service delivery differences do not result in geographical variation in the quality or quantity of the intervention. Targeted action and interventions aimed at improving vaccine uptake have been limited within the wider routine immunisation schedule [23]. We share Crocker-Buque et al.’s recommendation that additional support is needed to implement strategies to reduce inequalities [23].

This is not to say that there were not a number of providers going to great lengths to improve DNA rates through offering appropriate clinic times, locations, parent communication, re-booking, and reminder systems. Furthermore, these findings need to be heavily considered in conjunction with the challenge posed by staffing and resourcing. Some commissioners extended team capacity by contracting additional vaccinators and administrative staff to facilitate delivery of the new pathway. However, this was not universally available due to limited funding and availability of BCG trained immunisers or appropriate vaccination clinic venues. Without the option of expanding team capacity absorbing the sizable administration burden (e.g., appointment booking, reminders, recall) associated with the new pathway was challenging and sometimes fell on the BGC vaccinators themselves.

Pillay et al. cited concerns about additional NHS workload, focused on laboratory processes [13]. Our findings go beyond this to identify the additional staffing and resourcing requirements to deliver the programme effectively. Ongoing monitoring and evaluation of this challenge is required, with adaptations made accordingly. While administration burden associated with the BCG vaccine has increased, this is common across other vaccination programmes. Crocker-Buque et al. found that almost two-thirds (59.7%) of time associated with delivery of the routine immunisation programme was spent on administrative tasks with significant variation on where this burden was felt dependent on the model of delivery; some models placing greater strain on clinical (as opposed to non-clinical) staff [23].

Understanding and implementing new appointments and data systems was initially challenging; some issues have since been resolved while others are ongoing. Notably, some providers (26%) were waiting for SCID results prior to scheduling BCG appointments, which introduces unnecessary delays [27]. Additionally, most providers were still struggling with identification and fail-safe provision for infants moving in or out of the area. The survey finding regarding eligible infants not being referred and inappropriate referrals warrants further investigation.

Making sense of the new pathway and putting the specification into practice was challenging, some felt that there could have been stronger provision of centralised guidance on how to implement the change while still devolving power and autonomy to local stakeholders. This was associated with variations in how the programme change was implemented. Variation in the implementation of vaccination programmes because of iterative, local interpretation and tailoring is a finding shared with another process evaluation conducted by Crocker-Buque et al. [23]. Management factors, such as leadership, performance management, and stress are all known to affect programme effectiveness [28].

Innovative and varied solutions were developed in response to challenges, and consequently some challenges identified following the programme change were reported to have been resolved. Information sharing and localized, self-driven standardization was essential, although the mechanisms for this were often informally driven. The importance of collaboration and effective cross-system working was highlighted ahead of the change [13], and this was reflected in our findings with the formation of new communities of practice. This is particularly interesting here, due to the new interdependence between the vaccination and screening programme teams. Enrolling people into the new communities of practice required encouragement by articulating how the incoming change was relevant to them. Fostering these communities of practice and sharing learning could enable challenges to be overcome on a larger scale and contribute to the effective development of the programme going forward. The tension between providing centralised procedures and local autonomy has been observed in the literature before; comparing localised models of delivery to enable comparison and adoption of efficient pathways has been one suggestion [23].

Participant’s sentiment towards iterative programme implementation learning centered on whether they perceived this as part of putting a change into practice or a lack of foresight and appropriate planning. Those who felt positively about the new pathway reported the value of improved data capture and accountability. Comparatively, those who were concerned about the change commented on the feasibility of the 28-day target, increased DNA rates, resource pressures, and the lack of certainty that the change would be permanent. Notably, the protection of vulnerable infants through SCID screening was not frequently mentioned as a benefit, despite this being the reason for the change. Ambivalence was also challenging, as commissioners needed providers engagement to implement the change. The use of NPT to capture the contextual workplace culture towards the proposed BCG pathway change takes a first step in this under researched area [23].

It is common that programme implementation becomes less strategic and more sporadic over time [23]. Further cross-system collaboration is needed to improve the implementation of the new BCG pathway in line with the findings of this evaluation and ensure ongoing strategic oversight, particularly in relation to quality improvement cycles and addressing inequalities in BCG coverage.

### To summarise, as indicated by the findings of this study, key areas of consideration for policy and practice are as follows

1. The need to address DNA rates with tailored interventions;
2. the shortfall in staffing and resources needed to absorb the increased administrative burden;
3. the adoption of streamlined appointment systems (i.e., booking appointments preemptively in advance of SCID results);
4. the need for improved failsafe strategies to identify infants who were eligible for BCG but have not received vaccination (particularly for infants moving in/out of area);
5. the reticence/ambivalence of some staff towards the change;
  - by responding to the four concerns identified (the feasibility/rationale of the 28-day target; DNA rates; resourcing; and service change permanence);
  - by leveraging/celebrating the value of the new pathway for BCG delivery (improved data capture, accountability, and eligibility identification) but also strengthening the narrative around the duty of care to protect infants with SCID.

### Strengths and limitations

A strength of this study was the mixed methods approach, where the survey data provided an overview, and the qualitative data provided an in-depth examination of how the change was implemented and experienced. While we acknowledge that the synergy between the survey and qualitative data may have been better were this conceptualised as a mixed methods study from the offset, using NPT as a framework was an appropriate approach to triangulating the data from these two workstreams.

We would also like to acknowledge the interpretive nature of NPT, which is defined as a flexible and dynamic framework, providing areas of inspiration while allowing scope for concepts to emerge from the data [29]. As a result, certain domains of the theory are more strongly represented than others keeping true to the original data while using the theory to stimulate analytical insights. It is posited by the creators that findings may not fit neatly within the parameters of the theory, as is the case with any framework; in this analysis due to the iterative nature of coherence building, it was difficult to distinguish and separately report reflexive monitoring as in reality these factors were operating in tandem. This does not represent a weakness of the analysis, but an empirical finding in its own right.

Several limitations were identified. The surveys and interviews were conducted approximately one year after the change, and therefore captured views and experiences of the programme at a particular snapshot in time. There were not enough interviews to compare responses across commissioners, providers and CHIS’, but it is a strength that views from these stakeholder groups were captured. The provider and commissioner surveys focused on distinct aspects of the change, so responses cannot be compared between these groups.

It should be noted that where potential reasons for DNA are explored in this manuscript, this is based on the interpretations and beliefs of healthcare workers rather than directly from parents themselves. Further work is planned to capture parent perspectives, understand inequalities in BCG vaccination coverage, and investigate the impact of the change on TB and BCGosis cases. Similarly, while many participants expressed a need for greater guidance on implementation and 26% were using sub-optimal booking systems, we are unable to deduce whether this was due to an absence of guidance or gaps in communication and knowledge brokering. Nonetheless, our findings illustrate the need to either produce further guidance or improve the dissemination of pre-existing resources.

## Conclusion

The new BCG pathway has created an effective structure for monitoring and managing the BCG vaccination programme, but further work is required to support delivery of the 28-day target and improve uptake rates. The discussion provides insights for policy and practice, which could address challenging implications of the new pathway and ongoing tensions between national guidance versus local autonomy. Any ongoing risk of delaying BCG vaccination for SCID screening could be heavily mitigated by optimizing the implementation of the programme change. These findings are relevant to England and other countries which currently (or may go onto) deliver a BCG vaccination programme alongside SCID screening.

This study contributes to our growing understanding of the implementation of vaccination programmes within England. Efforts should be made to celebrate reform and promote a tolerant attitude towards service development. This requires a compromise between commissioners and service providers. A willingness from commissioners to proactively explore unintended implications of programme changes and provide appropriate guidance, alongside a willingness from providers to work iteratively within their locality where challenges could not be foreseen. When implementing new national vaccination pathways, stakeholder collaboration is essential for addressing challenges, harnessing benefits and sharing learning.

### Electronic supplementary material

Below is the link to the electronic supplementary material.

Supplementary Material 1

Supplementary Material 2

Supplementary Material 3

Supplementary Material 4

Supplementary Material 5

Supplementary Material 6

## Data Availability

The datasets generated and/or analysed during the current study are not publicly available due to their containing information that could compromise the privacy of research participants but are available from the corresponding author on reasonable request.

## References

[1] UK Health Security Agency. TB incidence and epidemiology in England, 2021. 2023 [cited 2023 07/07/2023]; https://www.gov.uk/government/publications/tuberculosis-in-england-2022-report-data-up-to-end-of-2021/tb-incidence-and-epidemiology-in-england-2021.

[2] World Health Organisation. Tuberculosis. 2023 [cited 2023 07/07/2023]; https://www.who.int/news-room/fact-sheets/detail/tuberculosis.

[3] NHS England and UK Health Security Agency. Tuberculosis (TB): action plan for England, 2021 to 2026. 2023 [cited 2023 11/07/2023]; https://www.gov.uk/government/publications/tuberculosis-tb-action-plan-for-england/tuberculosis-tb-action-plan-for-england-2021-to-2026.

[4] UK Health Security Agency. Tuberculosis: the green book, Chap. 32. 2018 [cited 2023 25/08/2023]; https://www.gov.uk/government/publications/tuberculosis-the-green-book-chapter-32.

[5] NHS. BCG vaccine for tuberculosis (TB) overview. 2019 [cited 2023 25/08/2023]; https://www.nhs.uk/conditions/vaccinations/bcg-tuberculosis-tb-vaccine/.

[6] UK Health Security Agency. Vaccine update: issue 327, April 2022, SCID, TB and BCG special edition. 2022; https://www.gov.uk/government/publications/vaccine-update-issue-327-may-2022-scid-tb-and-bcg-special-edition/vaccine-update-issue-327-april-2022-scid-tb-and-bcg-special-edition.

[7] Gennery AR, Worth A. Severe combined immunodeficiency: newborn screening and the BCG vaccination. Archives of Disease in Childhood; 2022. 10.1136/archdischild-2022-324682 35973752

[8] Nightingale R, Cavanagh C, Gennery AR (2021). Evaluation of newborn screening for severe combined immunodeficiency (SCID). Br J Gen Pract.

[9] Marshall J. *UK NSC public consultation: screening for severe combined immunodeficiency (SCID)*. 2017 [cited 2023 10/10/2023]; https://phescreening.blog.gov.uk/2017/10/13/uk-nsc-public-consultation-screening-for-severe-combined-immunodeficiency-scid/.

[10] Marciano BE (2014). BCG vaccination in patients with severe combined immunodeficiency: complications, risks, and vaccination policies. J Allergy Clin Immunol.

[11] UK Health Security Agency. BCG patient flowchart (UK Health Security Agency gateway number: 2021366. ANNB– BCG flowchart one. Product code: BCGA). 2021 07/07/2023]; https://assets.publishing.service.gov.uk/government/uploads/system/uploads/attachment_data/file/1039224/UKHSA-12101-BCG-patient-flowchart_Dec21.pdf.

[12] Oddie SJ, Mactler H (2022). Immunodeficiency screening: is the disruption is the BCG programme really warranted?. Arch Dis Child.

[13] Pillay T, Toldi G, Hussain A et al. Neonatal BCG: a time for change. Archives Disease Child– Educ Pract, 2022. Published Online First. 10.1136/archdischild-2021-323239 PMC10850640 36008111

[14] Elliman D. Study Design for an evaluation of Newborn Screening for SCID in the UK. Int J Neonatal Screen, 2022. 8(1). 10.3390/ijns8010004 PMC8788435 35076461

[15] May C, Cummings A, Girling M et al. Using normalization process theory in feasibility studies and process evaluations of complex healthcare interventions: a systematic review. Implement Sci, 2018. 13(1). 10.1186/s13012-018-0758-1 PMC5992634 29879986

[16] NHS England. What is commissioning?. 2023 06/09/2023]; https://www.england.nhs.uk/commissioning/what-is-commissioning/.

[17] NHS. NHS Data Model and Dictionary: Health Care Provider. 2023 06/09/2023]; https://www.datadictionary.nhs.uk/nhs_business_definitions/health_care_provider.html.

[18] InHealth. *Child Health Information Service (CHIS)*. 2023 [cited 2023 07/09/2023]; https://www.inhealth-intelligence.com/child-health-information-service/.

[19] Gale N, Heath G, Cameron E et al. Using the framework method for the analysis of qualitative data in multi-disciplinary health research. BMC Med Res Methodol, 2013. 13. 10.1186/1471-2288-13-117 PMC3848812 24047204

[20] Srivastava A, Thomson SB (2009). Framework analysis: a qualitative methodology for applied policy research. JOAAG.

[21] Pujolar G, Oliver-Anglès A, Vargas I, Vázquez ML. Changes in Access to Health Services during the COVID-19 pandemic: a scoping review. Int J Environ Res Public Health, 2022. 19(3). 10.3390/ijerph19031749 PMC8834942 35162772

[22] Spencer N, Markham W, Johnson S et al. The impact of COVID-19 pandemic on Inequity in Routine Childhood Vaccination Coverage: a systematic review. Vaccines, 2022. 10(7). 10.3390/vaccines10071013 PMC9321080 35891177

[23] Crocker-Buque T, Edelstein M, Mounier-Jack S (2018). A process evaluation of how the routine vaccination programme is implemented at GP practices in England. Implement Sci.

[24] Campbell H, Edwards A, Letley L (2017). Changing attitudes to childhood immunisation in English parents. Vaccine.

[25] Bedford HE, Elliman DAC (2020). Child and adolescent immunisation in the UK: current issues. Paediactrics Child Health.

[26] Smith D, Newton P (2016). Structural barriers to measles, mumps and rubella (MMR) immunisation uptake in Gypsy, Roma and Traveller communities in the United Kingdom. Crit Public Health.

[27] UK Health Security Agency. Guidance: Changing the timing of the neonatal BCG immunisation programme to a 28 day immunisation programme: effective from 1 September 2021. 2021 [cited 2023 10/08/2023]; https://www.gov.uk/government/publications/bcg-vaccine-information-on-the-28-day-immunisation-programme/changing-the-timing-of-the-neonatal-bcg-immunisation-programme-to-a-28-day-immunisation-programme-effective-from-1-september-2021.

[28] Michie S, West MA (2004). Managing people and performance: an evidence based framework applied to health service organizations. Int J Manage Reviews.

[29] May C, Rapley T, Mair FS et al. *Normalisation Process Theory On-line Users’ Manual, Toolkit and NoMAD instrument* 2015 [cited. 2023; http://www.normalizationprocess.org.

