# Supplementary material for: A new neonatal BCG vaccination pathway in England: a mixed methods evaluation of its implementation
Source: BMC Public Health. 2024 Apr 26;24:1175. doi: 10.1186/s12889-024-18586-8 (PMC11046867; doi:10.1186/s12889-024-18586-8)
Supplement: Supplementary file 1 — Supplementary Material 1 [file 12889_2024_18586_MOESM1_ESM.pdf]

Interview Topic Guide

BCG vaccine service providers

Date of interview: ________________________ Interviewer name:____________________________

Interviewee's ID number: __________________

Interviewees job title: ____

1. **Background information**
    - Please tell me about your current role and responsibilities for the NHS neonatal BCG vaccination programme.
    - Team (who you work with), accountability.
    - How long have you been involved in delivering the BCG vaccine programme?
        - Is this pre-change or not?
    - Any involvement in SCID screening.

2. Coherence – making sense of the reasons for the new BCG pathway

- What do you think about the new BCG pathway? Do you think it is a good idea? What is good/bad about it?
- What training and information was available about the new BCG pathway, when was it available, what did it cover, how did it help?
- What challenges does the new BCG pathway provide? What are the potential benefits?
- Are these views shared across the organisation?

3. Cognitive participation – buy-in to the new BCG pathway (commitment)

- What is the buy-in like from staff/the organisation? Are staff committed to the change?
- Why do you think that is?
- Any sub(groups) with specific issues?
- Did your department/practice initiate any actions to get staff on-board/increase buy-in and involvement? E.g., champions or key person in organisation; peer support groups? Were you involved in these activities?
- If not do you think it would have been a good idea?
- Did you face any challenges/what helped; What do you think is needed?
- In your experience, how are parents responding to BCG vaccination and the new pathway?
    - BCG vaccination perceptions and behaviours
    - Reasons for the change in vaccination timing
    - Experience of outreach and communication with parents
    - Any inequalities in uptake between different population groups

4. Collective action – putting the new BCG pathway into action.

- Process:
- How is the new NHS neonatal BCG pathway being implemented in practice? What are the models being used by service providers?
    - Location of service
    - Time / date of vaccination
    - Who vaccinates
    - Notification of service users
- What are your experiences/views of accessing data on BCG eligibility and SCID screening results?
    - Accessibility of data/results
    - Effectiveness of IT systems, including CHIS and S4N
    - Data sharing processes
    - Confidence in evaluating results and taking relevant action
- Could you walk me through the guidance or training you received prior/during the implementation of the new pathway?
    - Timeliness; accessibility; usefulness; feedback; support.
    - Is there any documentation on this available (e.g. staff training manuals, training evaluation, new job descriptions, engagement with colleges on the training, future training plans)? Can you share this with us?
- What are your views/experiences of implementing the change?
    - Time to prepare
    - Implications for practice
- What went well? What factors facilitated the implementation of the new pathway?
    - Political/social
    - Administrative, logistical
    - Regulatory
- What was difficult? What barriers made it difficult to implement the new pathway? Has anything been put in place to address these concerns/challenges? If so could you send us some information on this?
    - Political/social
    - Administrative, logistical
    - Regulatory
- Impact:
    - Has the new BCG pathway made your life easier or more difficult in the long-term?
    - Impact of the new BCG pathway on your morale/wellbeing (e.g., confidence, impact on clinical risk).
    - Impact of the new BCG pathway on workflow and working practices.
    - Impact of the new BCG pathway on your workload.
- What are the key issues affecting BCG vaccination uptake in your area? How are vaccine inequalities assessed? How are they addressed?
    - Underserved populations
    - Vaccine confidence
    - Accessibility of services
    - Outreach and communication activities

- How have the new service performance indicators been integrated (how are they working)? Have you observed any changes to service performance due to the programme changes or due to the COVID-19 pandemic?
    - Any unintended outcomes (positive/negative)?

5. Reflexive monitoring: appraisals of remote consulting.

- What have been the key learning points?
    - Lessons learnt; if starting from the beginning anything which you would do differently;
    - Any changes which need to happen locally to make it work better;
    - Advice you would give to another site about to start their task of implementing the new pathway.
- What are the key considerations for the future?
- How have staff been able to feed back issues which need improvements? Have you discussed any issues arising? Has your unit/organisation made changes based on these discussions?
    - Who is involved in collaboration?
    - Communication pathways?
    - Nature of collaboration?

6. Any other issues

Any other issues? Is there anything important that I have not asked you about?
(Reminder of prior questions. You mentioned X, Y Z. Would you be able to share that information with us?).
Thank them for their time and check preferences regarding receipt of summary of study findings.
