# Supplementary material for: A new neonatal BCG vaccination pathway in England: a mixed methods evaluation of its implementation
Source: BMC Public Health. 2024 Apr 26;24:1175. doi: 10.1186/s12889-024-18586-8 (PMC11046867; doi:10.1186/s12889-024-18586-8)
Supplement: Supplementary file 2 — Supplementary Material 2 [file 12889_2024_18586_MOESM2_ESM.pdf]

Interview Topic Guide

BCG vaccine programme commissioners/CHIS

Date of interview: ________________________ Interviewer name:__________________________

Interviewee's ID number: __________________

Interviewees role/job title: ____

1. **Background information**

- Please tell me about your current role and responsibilities for the NHS neonatal BCG vaccination programme.
- Team (who you work with), accountability.
- How long have you been involved in BCG vaccine commissioning?
  - Is this pre-change or not?
- Any involvement in commissioning SCID screening.

2. Coherence – making sense of the reasons for the new BCG pathway

- What do you think about the new BCG pathway? Do you think it is a good idea? What is good/bad about it?
- What training, information materials was available about the new BCG pathway? When was this offered, what was covered, how did it help?
- What challenges does the new BCG pathway provide? What are the benefits you hoped the new BCG pathway would achieve?
- Are these views shared across the organisation?

3. Cognitive participation – buy-in to the new BCG pathway (commitment)

- What was the buy-in like from staff/ organisation? Are staff committed to the change?
- Why do you think that is?
- Any sub(groups) with specific issues?
  - Did your department/practice initiate any actions to get staff on-board/increase buy-in and involvement? E.g. champions or key person in organisation; training; peer support groups? Were you involved in these activities?
  - If not do you think it would have been a good idea?
  - Did you face any challenges/what helped; good or bad aspects of the training? What do you think is needed?

4. Collective action – putting the new BCG pathway into action.

- Process:
- What changes have been made to the NHS neonatal BGC vaccine programme commissioning process because of the new pathway?
  - Co-ordination/operation of programme:
    Involvement of additional people/organisations/systems (CHIS, service providers)
  - Communication between organisations

    - Cost, human resources
    - Additional guidance or training for providers

- How is the new NHS neonatal BCG pathway being implemented in practice? Which implementation models are being commissioned and why?
    - Location of service
    - Date/time of vaccination
    - Who vaccinates
    - Notification of service users

- What are your views/experiences of implementing the change?
    - Sentiment to the change
    - Time to prepare
    - Implications for practice

- What went well? What factors facilitated the implementation of the new pathway?
    - Political/social
    - Administrative, logistical
    - Regulatory

- What was difficult? What barriers made it difficult to implement the new pathway? Has anything been put in place to address these concerns/challenges? If so could you send us some information on this?
    - Political/social
    - Administrative, logistical
    - Regulatory
- Impact:
    - Has the new BCG pathway made your life easier or more difficult in the long-term?
    - Impact of the new BCG pathway on your morale/wellbeing (e.g., confidence, impact on clinical risk).
    - Impact of the new BCG pathway on workflow and working practices.
    - Impact of the new BCG pathway on your workload.
    - Training, learning opportunities, support for staff?
- What are the key issues affecting BCG vaccination uptake in your area? How are vaccine inequalities assessed? How are they addressed?
    - Underserved populations
    - Vaccine confidence
    - Accessibility of services
    - Outreach and communication activities
- How have the new service performance indicators been integrated (how are they working)? Have you observed any changes to service performance due to the programme changes or due to the COVID-19 pandemic?
    - Any unintended outcomes (positive/negative)?

5. Reflexive monitoring: appraisals of new models and practice

- What have been the key learning points?
    - Lessons learnt; if starting from the beginning anything which you would do differently;
    - Any changes which need to happen locally to make it work better;
    - Advice you would give to another site about to start their task of implementing the new pathway.
- What are the key considerations for the future?
- How have staff been able to feed back issues which need improvements? Have you discussed any issues arising during meetings? Has your unit made changes based on these discussions?
    - Who is involved in collaboration?
    - Communication pathways?
    - Nature of collaboration?

6. Any other issues

Any other issues? Is there anything important that I have not asked you about?

(Reminder of prior questions. You mentioned X, Y Z. Would you be able to share that information with us?).

Thank them for their time and check preferences regarding receipt of summary of study findings.
