# Supplementary material for: A new neonatal BCG vaccination pathway in England: a mixed methods evaluation of its implementation
Source: BMC Public Health. 2024 Apr 26;24:1175. doi: 10.1186/s12889-024-18586-8 (PMC11046867; doi:10.1186/s12889-024-18586-8)
Supplement: Supplementary file 3 — Supplementary Material 3 [file 12889_2024_18586_MOESM3_ESM.pdf]

UK Health Security Agency

BCG immunisation programme change evaluation - NHSE Regions Survey (Providers)

Introduction

The United Kingdom Health Security Agency (UKHSA), London School of Hygiene and Tropical Medicine, University of Bristol and NHS England are undertaking an evaluation of the implementation of the S7A BCG neonatal vaccination patient pathway for children under the age of 1 year. This follows the change in the timing of the BCG vaccination offer due to the introduction of screening for Severe Combined Immunodeficiency (SCID) syndrome.

This survey is one of many strands of work that when combined will help us understand how the pathway is being implemented, associated barriers and facilitators, and examples of good practice. This is vital in minimising negative effects and harnessing the positives to further improve the quality of the programme going forward.

Please can you ensure that this survey is completed by either an NHS regional public health commissioning lead/screening and immunisation lead for each individual provider in your NHS region, or that the survey link is sent to each individual provider directly for completion. Please can you ensure that this survey is completed no later than 30/11/2022. This survey can be closed during completion, and responses will be saved. You will be able to return to the first uncompleted page of the survey when you click on the survey link, but will not be able to update answers once the survey has been submitted.

The answers given will be kept in strict confidence and will be held and processed securely in line with the Data Protection Act 2018 and UKHSA information governance policies and procedures. Reporting of the findings will be anonymised so that readers won’t be able to identify individual responses.

If you have any queries about this survey, please contact the UKHSA immunisation team via email: immunisation@ukhsa.gov.uk

1. What is your name?*

2. What is your role?*

3. What is your region?*

- ☐ East of England
- ☐ London

☐ Midlands
☐ North East and Yorkshire
☐ North West
☐ South East
☐ South West

UK Health Security Agency

BCG immunisation programme change evaluation - NHSE Regions Survey (Providers)

Provider Overview

4. Provider name*

[ ]

5. Provider type*

-- Please Select --

6. Was this provider commissioned to deliver the BCG vaccination programme in your area **prior to** 1st September 2021?*

-- Please Select --

UK Health Security Agency

BCG immunisation programme change evaluation - NHSE Regions Survey (Providers)

Geographical coverage and accessibility of clinics

7. Please select all of the local authorities covered by this provider in your region*

Please select all local authorities covered by this provider.

- ☐ North East: County Durham
- ☐ North East: Darlington
- ☐ North East: Gateshead
- ☐ North East: Hartlepool
- ☐ North East: Middlesbrough
- ☐ North East: Newcastle upon Tyne
- ☐ North East: North Tyneside
- ☐ North East: Northumberland
- ☐ North East: Redcar and Cleveland
- ☐ North East: South Tyneside
- ☐ North East: Stockton-on-Tees
- ☐ North East: Sunderland
- ☐ North West: Blackburn with Darwen
- ☐ North West: Blackpool
- ☐ North West: Bolton
- ☐ North West: Bury
- ☐ North West: Cheshire East
- ☐ North West: Cheshire West and Chester
- ☐ North West: Cumbria
- ☐ North West: Halton
- ☐ North West: Knowsley
- ☐ North West: Lancashire
- ☐ North West: Liverpool
- ☐ North West: Manchester
- ☐ North West: Oldham
- ☐ North West: Rochdale
- ☐ North West: Salford
- ☐ North West: Sefton
- ☐ North West: St. Helens
- ☐ North West: Stockport
- ☐ North West: Tameside
- ☐ North West: Trafford
- ☐ North West: Warrington
- ☐ North West: Wigan
- ☐ North West: Wirral
- ☐ Yorkshire and Humber: Barnsley
- ☐ Yorkshire and Humber: Bradford
- ☐ Yorkshire and Humber: Calderdale
- ☐ Yorkshire and Humber: Doncaster
- ☐ Yorkshire and Humber: East Riding of Yorkshire
- ☐ Yorkshire and Humber: Kingston upon Hull
- ☐ Yorkshire and Humber: Kirklees
- ☐ Yorkshire and Humber: Leeds
- ☐ Yorkshire and Humber: North East Lincolnshire
- ☐ Yorkshire and Humber: North Lincolnshire

- ☐ Yorkshire and Humber: North Yorkshire
- ☐ Yorkshire and Humber: Rotherham
- ☐ Yorkshire and Humber: Sheffield
- ☐ Yorkshire and Humber: Wakefield
- ☐ Yorkshire and Humber: York
- ☐ East Midlands: Derby
- ☐ East Midlands: Derbyshire
- ☐ East Midlands: Leicester
- ☐ East Midlands: Leicestershire
- ☐ East Midlands: Lincolnshire
- ☐ East Midlands: Nottingham
- ☐ East Midlands: Nottinghamshire
- ☐ East Midlands: North Northamptonshire
- ☐ East Midlands: West Northamptonshire
- ☐ East Midlands: Rutland
- ☐ West Midlands: Birmingham
- ☐ West Midlands: Coventry
- ☐ West Midlands: Dudley
- ☐ West Midlands: Herefordshire
- ☐ West Midlands: Sandwell
- ☐ West Midlands: Shropshire
- ☐ West Midlands: Solihull
- ☐ West Midlands: Staffordshire
- ☐ West Midlands: Stoke-on-Trent
- ☐ West Midlands: Telford and Wrekin
- ☐ West Midlands: Walsall
- ☐ West Midlands: Warwickshire
- ☐ West Midlands: Wolverhampton
- ☐ West Midlands: Worcestershire
- ☐ East of England: Bedford
- ☐ East of England: Cambridgeshire
- ☐ East of England: Central Bedfordshire
- ☐ East of England: Essex
- ☐ East of England: Hertfordshire
- ☐ East of England: Luton
- ☐ East of England: Norfolk
- ☐ East of England: Peterborough
- ☐ East of England: Southend-on-Sea
- ☐ East of England: Suffolk
- ☐ East of England: Thurrock
- ☐ London: Barking and Dagenham
- ☐ London: Barnet
- ☐ London: Bexley
- ☐ London: Brent
- ☐ London: Bromley
- ☐ London: Camden
- ☐ London: City of London
- ☐ London: Croydon

- ☐ London: Ealing
- ☐ London: Enfield
- ☐ London: Greenwich
- ☐ London: Hackney
- ☐ London: Hammersmith and Fulham
- ☐ London: Haringey
- ☐ London: Harrow
- ☐ London: Havering
- ☐ London: Hillingdon
- ☐ London: Hounslow
- ☐ London: Islington
- ☐ London: Kensington and Chelsea
- ☐ London: Kingston upon Thames
- ☐ London: Lambeth
- ☐ London: Lewisham
- ☐ London: Merton
- ☐ London: Newham
- ☐ London: Redbridge
- ☐ London: Richmond upon Thames
- ☐ London: Southwark
- ☐ London: Sutton
- ☐ London: Tower Hamlets
- ☐ London: Waltham Forest
- ☐ London: Wandsworth
- ☐ London: Westminster
- ☐ South East: Bracknell Forest
- ☐ South East: Brighton and Hove
- ☐ South East: Buckinghamshire
- ☐ South East: East Sussex
- ☐ South East: Hampshire
- ☐ South East: Isle of Wight
- ☐ South East: Kent
- ☐ South East: Medway
- ☐ South East: Milton Keynes
- ☐ South East: Oxfordshire
- ☐ South East: Portsmouth
- ☐ South East: Reading
- ☐ South East: Slough
- ☐ South East: Southampton
- ☐ South East: Surrey
- ☐ South East: West Berkshire
- ☐ South East: West Sussex
- ☐ South East: Windsor and Maidenhead
- ☐ South East: Wokingham
- ☐ South West: Bath and North East Somerset
- ☐ South West: Bournemouth, Christchurch and Poole
- ☐ South West: Bristol
- ☐ South West: Cornwall

- ☐ South West: Devon
- ☐ South West: Dorset
- ☐ South West: Gloucestershire
- ☐ South West: Isles of Scilly
- ☐ South West: North Somerset
- ☐ South West: Plymouth
- ☐ South West: Somerset
- ☐ South West: South Gloucestershire
- ☐ South West: Swindon
- ☐ South West: Torbay
- ☐ South West: Wiltshire

8. How many BCG vaccination sites are available on average per local authority covered?*

   -- Please Select --

9. What is the maximum distance patients need to travel to access clinics? *

   -- Please Select --

10. How frequently are the clinics offered at the different sites?*

    -- Please Select --

11. When are clinics offered by this provider?*

    - ☐ Daytime only clinics
    - ☐ Evening clinics
    - ☐ Weekend clinics
    - ☐ Other, please specify

12. Has an assessment been made of the accessibility of clinic sites and dates/times?*

    -- Please Select --

13. If yes, what was the outcome?*

UK Health Security Agency

BCG immunisation programme change evaluation - NHSE Regions Survey (Providers)

Service Components

14. Who is responsible for assessing neonates' BCG eligibility status and ensuring this data is sent to CHIS?*

15. Is the process for assessing neonates' BCG eligibility status and making sure this data is sent to CHIS generally working well? *

-- Please Select --

16. Please outline any challenges identified with assessing neonates' BCG eligibility status and ensuring this data is sent to CHIS*

17. Is S4N being used?*

-- Please Select --

18. Is the referral process for eligible babies to the BCG immunisation provider working well?*

-- Please Select --

19. If no, what are the challenges?

20. When are the BCG vaccination appointments usually booked?*

-- Please Select --

21. Which clinician is usually responsible for assessing the outcome of SCID screening for babies eligible for BCG vaccination?*

-- Please Select --

22. Is the current SCID screening outcome assessment process working well? *

-- Please Select --

23. Please outline any challenges identified with the current SCID screening outcome assessment process

24. Have any BCG vaccination appointments had to be rescheduled due to challenges accessing SCID results prior to the scheduled appointment?*

-- Please Select --

25. If yes, please provide further details *

26. When a baby has a result 'SCID suspected', how do you find out the outcome of the immunology assessment?*

- ☐ From a GP
- ☐ From the immunology service
- ☐ From the parents
- ☐ Other, please specify

27. Does a BCG appointment ever have to be delayed due to the method of finding out the SCID screening result?*

-- Please Select --

UK Health Security Agency

BCG immunisation programme change evaluation - NHSE Regions Survey (Providers)

Call and Recall

28. How is the first vaccination invitation sent?*

- ☐ Phone
- ☐ Text
- ☐ Letter
- ☐ Other, please specify

29. How many reminder invitations are sent?*

-- Please Select --

30. How are the reminder vaccination invitations sent?*

- ☐ Phone
- ☐ Text
- ☐ Letter
- ☐ Other, please specify

31. What are the DNA rates for the service?*

-- Please Select --

UK Health Security Agency

BCG immunisation programme change evaluation - NHSE Regions Survey (Providers)

Information Tailored to the Population Served

32. Are parents or guardians given a copy of the UKHSA BCG vaccine leaflet?*

-- Please Select --

33. When are parents or guardians given the UKHSA leaflet?*

-- Please Select --

34. Are parents or guardians given a local leaflet? *

-- Please Select --

35. When are parents or guardians given the local leaflet?*

-- Please Select --

36. Is the local leaflet available in multiple languages?*

-- Please Select --

37. What languages is the local leaflet available in?*

- ☐ Arabic
- ☐ Bengali
- ☐ Cantonese
- ☐ Filipino
- ☐ French
- ☐ German
- ☐ Gujarati
- ☐ Italian
- ☐ Lithuanian
- ☐ Mandarin
- ☐ Persian/Farsi

☐ Polish
☐ Portuguese
☐ Punjabi
☐ Romanian
☐ Somali
☐ Spanish
☐ Tamil
☐ Turkish
☐ Urdu
☐ Other, please specify

38. Are parents offered any additional information about the BCG vaccine?*

☐ Yes
☐ No
☐ Sometimes

39. What additional information is provided? *

☐ Clinical advice
☐ Website links
☐ Other, please specify

40. When are parents or guardians given the additional information?*

-- Please Select --

41. Is the additional information available in multiple languages?*

-- Please Select --

42. What languages is the additional information available in?*

☐ Arabic
☐ Bengali
☐ Cantonese
☐ Filipino
☐ French
☐ German
☐ Gujarati
☐ Italian
☐ Lithuanian

- ☐ Mandarin
- ☐ Persian/Farsi
- ☐ Polish
- ☐ Portuguese
- ☐ Punjabi
- ☐ Romanian
- ☐ Somali
- ☐ Spanish
- ☐ Tamil
- ☐ Turkish
- ☐ Urdu
- ☐ Other, please specify

43. Any further comments?
