# Supplementary material for: A new neonatal BCG vaccination pathway in England: a mixed methods evaluation of its implementation
Source: BMC Public Health. 2024 Apr 26;24:1175. doi: 10.1186/s12889-024-18586-8 (PMC11046867; doi:10.1186/s12889-024-18586-8)
Supplement: Supplementary file 4 — Supplementary Material 4 [file 12889_2024_18586_MOESM4_ESM.pdf]

UK Health Security Agency

BCG immunisation programme change evaluation - NHSE Regions Survey

Introduction

The United Kingdom Health Security Agency (UKHSA), London School of Hygiene and Tropical Medicine, University of Bristol and NHS England are undertaking an evaluation of the implementation of the S7A BCG neonatal vaccination patient pathway for children under the age of 1 year. This follows the change in the timing of the BCG vaccination offer due to the introduction of screening for Severe Combined Immunodeficiency (SCID) syndrome.

This survey is one of many strands of work that when combined will help us understand how the pathway is being implemented, associated barriers and facilitators, and examples of good practice. This is vital in minimising negative effects and harnessing the positives to further improve the quality of the programme going forward.

Please can you ensure that this survey is completed by an NHS regional public health commissioning lead or screening and immunisation lead no later than 30/11/2022. This survey can be closed during completion, and responses will be saved. You will be able to return to the first uncompleted page of the survey when you click on the survey link, but will not be able to update answers once the survey has been submitted.

The answers given will be kept in strict confidence and will be held and processed securely in line with the Data Protection Act 2018 and UKHSA information governance policies and procedures. Reporting of the findings will be anonymised so that readers won't be able to identify individual responses.

If you have any queries about this survey, please contact the UKHSA immunisation team via email: immunisation@ukhsa.gov.uk

1. What is your name?*

2. What is your role?*

3. What is your region?*
   - ☐ East of England
   - ☐ London
   - ☐ Midlands
   - ☐ North East and Yorkshire
   - ☐ North West
   - ☐ South East
   - ☐ South West

UK Health Security Agency

BCG immunisation programme change evaluation - NHSE Regions Survey

Provider Overview

4. How many providers were commissioned to deliver the BCG vaccination programme in your area **prior to** 1st

September 2021?*

-- Please Select --

5. What types of providers were commissioned to deliver the BCG vaccination programme in your area **prior to** 1st September 2021?*

Select all that apply

- ☐ Hospital trust
- ☐ Maternity service
- ☐ Community trust
- ☐ Clinical commissioning group
- ☐ Specialist BCG clinic
- ☐ TB service
- ☐ Outpatient clinic
- ☐ GP
- ☐ Other, please specify

6. How many providers have been commissioned to deliver the BCG vaccination programme in your area **since** 1st September 2021?*

-- Please Select --

7. What types of providers have been commissioned to deliver the BCG vaccination programme in your area **since** 1st September 2021?*

Select all that apply

- ☐ Hospital trust
- ☐ Maternity service
- ☐ Community trust
- ☐ Clinical commissioning group
- ☐ Specialist BCG clinic
- ☐ TB service
- ☐ Outpatient clinic
- ☐ GP
- ☐ Other, please specify

UK Health Security Agency

BCG immunisation programme change evaluation - NHSE Regions Survey

Child Health Information Services (CHIS)

8. How many CHIS providers are there in your NHS region?*

-- Please Select --

9. Is the BCG eligibility data being transferred through S4N alongside the NIPE (Newborn and Infant Physical Examination) information?*

-- Please Select --

10. If no, what alternative arrangement is in place?*

11. Have you assessed the completion of eligibility information on CHIS since the implementation of the information standard in September 2021?*

https://assets.publishing.service.gov.uk/government/uploads/system/uploads/attachment_data/file/1005937/Changing_the_timing_of__the_neonatal_BCG_immunisation_programme_letter_July_2021.pdf

-- Please Select --

12. If yes, what is the percentage:*

a) Eligible

b) Not eligible

c) Not known

13. What do you think the completion of BCG eligibility information on CHIS is in your area?*

-- Please Select --

14. What actions are being taken to **monitor** the completion of BCG eligibility information on CHIS in your area?*

15. What actions are being taken to **improve** the completion of BCG eligibility information on CHIS in your area?

UK Health Security Agency

BCG immunisation programme change evaluation - NHSE Regions Survey

COVID-19 Pandemic

16. Did the COVID-19 pandemic impact your region's ability to **prepare** for the change to the BCG vaccination programme in September 2021?*

-- Please Select --

17. How did the COVID-19 pandemic impact your region's ability to **prepare** for the change to the BCG vaccination programme?*

18. Did the COVID-19 pandemic impact your region's ability to **implement** the change to the BCG vaccination programme?*

-- Please Select --

19. How did the COVID-19 pandemic impact your region's ability to **implement** the change to the BCG vaccination programme?*

20. Was the delivery of the BCG vaccination programme interrupted in all or part of your area during the COVID-19 pandemic? *

-- Please Select --

21. How long was the BCG vaccination programme interrupted for during the COVID-19 pandemic?*

22. What was the **cause** of the BCG vaccination programme interruption during the COVID-19 pandemic?*

23. What was the **impact** of the BCG vaccination programme interruption during the COVID-19 pandemic?*

UK Health Security Agency

BCG immunisation programme change evaluation - NHSE Regions Survey

Change Implementation

24. How would you rate the implementation of the new BCG vaccination programme in your region? *

-- Please Select --

25. Please outline the reasons for the above rating*

26. Have there been any challenges in implementing the change to the BCG vaccination programme?*

-- Please Select --

27. What were the challenges?

- [ ] Complex changes
- [ ] Providers were not available
- [ ] Providers were not on board with the change
- [ ] Financial issues
- [ ] Stakeholder engagement

☐ Other, please specify

28. Please provide any further details on the challenges identified*

29. Have there been any benefits in implementing the change to the BCG vaccination programme?*

-- Please Select --

30. Please provide further details on the benefits identified*

UK Health Security Agency

BCG immunisation programme change evaluation - NHSE Regions Survey

Training

31. Are you aware of the UKHSA training resources and information for health professionals?*

https://www.gov.uk/government/collections/bcg-vaccination-programme

-- Please Select --

32. How would you rate these resources?*

-- Please Select --

33. Please provide further details on the rating given

34. Were you able to access training for any new providers that were onboarded? *

-- Please Select --

UK Health Security Agency

BCG immunisation programme change evaluation - NHSE Regions Survey

BCG Vaccination Uptake

35. Do you know what the BCG vaccine uptake percentage has been amongst the eligible population between 1st September 2021 and 30th June 2022 in your region?*

-- Please Select --

36. What percentage of vaccines have been delivered at or before 28 days of age between 1st September 2021 and 30th June 2022? *

-- Please Select --

37. What do you think are the key challenges for achieving higher uptake in your area?

- ☐ Clinic accessibility
- ☐ Tailored information
- ☐ Appointment invites and reminders
- ☐ Available appointment times
- ☐ Other, please specify

UK Health Security Agency

BCG immunisation programme change evaluation - NHSE Regions Survey

Case studies

38. Please share any examples of good practice related to improving data capture for BCG eligibility on CHIS, improving BCG uptake, and reducing inequalities

39. Have the above examples of good practice been evaluated?
