# Supplementary material for: A new neonatal BCG vaccination pathway in England: a mixed methods evaluation of its implementation
Source: BMC Public Health. 2024 Apr 26;24:1175. doi: 10.1186/s12889-024-18586-8 (PMC11046867; doi:10.1186/s12889-024-18586-8)
Supplement: Supplementary file 5 — Supplementary Material 5 [file 12889_2024_18586_MOESM5_ESM.pdf]

UK Health Security Agency

Evaluation Survey: Commissioner Data

Region and Role

Region

Eleven responses were received to the commissioner survey, with responses received from each of the seven NHS regions. Where multiple responses were received from a particular region, this was due to there being different commissioning areas within a region.

**Characteristic**	**N = 11**[1]
respondent region	
South East	3 (27%)
Midlands	2 (18%)
North East and Yorkshire	2 (18%)
East of England	1 (9.1%)
London	1 (9.1%)

[1] n (%)

Characteristic	N = 11[1]
North West	1 (9.1%)
South West	1 (9.1%)

[1] n (%)

Role

The most common respondent role was Screening and Immunisation Manager (45%), followed by Screening and Immunisation Lead (18%)

Characteristic	N = 11[1]
respondent role	
Screening and Immunisation Manager	5 (45%)
Screening and Immunisation Lead	2 (18%)
Immunisation Commissioning Manager	1 (9.1%)
Public Health Programmes Manager	1 (9.1%)
Screening and Immunisation Coordinator	1 (9.1%)
Senior Contract Performance & Commissioning Project Manager	1 (9.1%)

[1] n (%)

Overview of Providers

Number of Providers Per Region

The number of providers have remained the same in all regions since the change to the BCG vaccination programme in September 2021, apart from in the East of England where the number of providers has decreased from 10+ to 2. The most common number of providers per region both before and after the change was 10+ (64% and 55% respectively).

Characteristic	N = 11[1]
number of providers per region prior to September 2021	
10+	7 (64%)
4	1 (9.1%)
5	1 (9.1%)
7	1 (9.1%)
8	1 (9.1%)

[1] n (%)

Characteristic	N = 11[1]
number of providers per region after September 2021	
10+	6 (55%)
2	1 (9.1%)
4	1 (9.1%)

[1] n (%)

Characteristic	N = 11[1]
5	1 (9.1%)
7	1 (9.1%)
8	1 (9.1%)

[1] n (%)

CHIS

Number of CHIS Providers

The number of CHIS providers in each region ranged from 1 to 5+. The most common number of CHIS providers was 3 providers (36%), followed by 5+ providers (27%). All of the responses reported that CHIS providers were using S4N*.

*The new Newborn and Physical Examination (NIPE) IT system, SMaRT4NIPE (S4N) went live on 1 April 2019.

Characteristic	N = 11[1]
number of CHIS providers	
1	2 (18%)
2	1 (9.1%)
3	4 (36%)
4	1 (9.1%)
5+	3 (27%)

[1] n (%)

BCG Eligibility on CHIS

The completion of BCG eligibility information on CHIS was 80%+ for five respondents (45%), whilst six (55%) responded that the completion of BCG eligibility information on CHIS was unknown.

To improve the recording of BCG eligibility on CHIS, commissioners suggested a mandatory field on S4N for recording BCG eligibility, encouraging communication between providers, working with stakeholders to ensure that patients moving into an area are recorded on CHIS, training to ensure that eligibility is correctly recorded, and ongoing monitoring. Other commissioners noted that the current process is working effectively.

**Characteristic**	**N = 11**[1]
completion of eligibility information on CHIS	
Unknown	6 (55%)
80%+	5 (45%)

[1] n (%)

COVID-19 Pandemic

COVID-19 Pandemic and Impact on BCG Vaccination Preperation and Implementation

The COVID-19 pandemic had an impact on 64% of respondents ability to prepare for or implement the change to the BCG vaccination programme. Four respondents (36%) indicated that

the COVID-19 pandemic did not have an impact on the ability to prepare for or implement the change to the BCG vaccination programme.

Characteristic	N = 11[1]
COVID-19 impact on BCG vaccination preperation	
COVID-19 had an impact on BCG vaccination preperation	7 (64%)
COVID-19 did not have an impact on BCG vaccination preperation	4 (36%)

[1] n (%)

Characteristic	N = 11[1]
COVID-19 impact on BCG vaccination implementation	
COVID-19 did not have an impact on BCG vaccination implementation	6 (55%)
COVID-19 had an impact on BCG vaccination implementation	5 (45%)

[1] n (%)

COVID-19 Pandemic Interruption

The COVID-19 pandemic caused an interruption to the delivery of the BCG vaccination programme for 5 (45%) respondents (South East (n=3), Midlands (n=1) and North East (n=1)).

Where the COVID-19 pandemic caused an interruption to the delivery of the BCG vaccination programme, this was due to staffing pressures and staff illness, families unable to attend

appointments due to illness or anxiety about COVID-19 exposure, and clinics being cancelled due to limiting footfall on wards.

The impact of the interruption to the delivery of the BCG vaccination programme due to COVID-19 was potentially lower vaccination uptake, delays to vaccination, and backlogs were created.

**Characteristic**	**N = 11**[1]
COVID-19 impact on BCG vaccination delivery	
COVID-19 did not interrupt the delivery of the BCG vaccination programme	6 (55%)
COVID-19 interrupted the delivery of the BCG vaccination programme	5 (45%)

[1] n (%)

Change Implementation

Implementation Rating

The ratings for the implementation of the new BCG vaccination programme were good (45%), fair (27%) and neutral (27%). There were no respondents that rated the implementation as either poor or excellent.

Reasons for lower ratings included providers struggling to meet the 28 day BCG vaccination target, increase in workload (particularly to setup new clinics), lack of additional funding and resources to deliver the programme, a short implementation timeline, and challenges with data reporting.

Reasons for higher ratings included good engagement from providers and strong leadership from commissioners, providers being compliant with the change, and providers successfully implementing the programme.

Characteristic	N = 11[1]
implementation rating	
Good	5 (45%)
Fair	3 (27%)
Neutral	3 (27%)

[1] n (%)

Implementation Challenges

All respondents (100%) noted that there had been implementation challenges, including:

- Difficulties meeting the 28 day target for BCG vaccination
- Lack of additional funding or resources to deliver the programme
- Issues around obtaining SCID results, and difficulties transferring data between organisations
- Additional admin support required to arrange and monitor appointments and reporting of KPIs
- Implementing a significant change during the pandemic when there were issues with sickness and workload
- Involvement of multiple stakeholders
- High DNA rates, as infants need to attend a separate appointment to receive the vaccination
- Short implementation time and late publication of guidance
- Challenges monitoring infants born out of region but who reside within the region, and infants born within the

region who reside out of the region
- Backlogs in delivery which had not been cleared prior to the introduction of the new specification
- Delays to BCG vaccine being administered and clinical risk involved
- In areas that are not part of the SCID pilot, providers challenged the relevance of the programme change

**Characteristic**	**N = 11**[1]
implementation challenges	
Implementation challenges identified	11 (100%)

[1] n (%)

Implementation Benefits

Eight respondents (73%) identified implementation benefits, including:

- More robust and consistent data being collected
- Meaningful data in terms of eligibility, delivery, uptake and coverage, which enables follow up action to be taken
- Providers are successfully providing the service according to national guidelines
- Better knowledge around identification of eligibility, and more infants recognised as eligible for vaccination
- Good relationships developed between providers and ICB’s
- More staff trained to deliver the service, which has improved resilience

**Characteristic**	**N = 11**[1]
implementation benefits	

[1] n (%)

Characteristic	N = 11[1]
Implementation benefits identified	8 (73%)
Implementation benefits not identified	3 (27%)

[1] n (%)

Training

UKHSA Training Resources

All respondents were aware of the UKHSA training resources and information for health professionals. The majority of respondents rated the training resources ‘Good’ (73%), whilst 18% rated the resources ‘Excellent’, and 9.1% rated the resources ‘Fair’.

Where further information was provided, respondents noted that the resources were comprehensive, thorough and helpful to clinical staff, and are used by providers. It was also noted that provision of national BCG training would be welcomed.

Characteristic	N = 11[1]
training resource rating	
Good	8 (73%)
Excellent	2 (18%)
Fair	1 (9.1%)

[1] n (%)

Accessing Training for New

Providers

Training for new providers could be accessed by 6 (55%) respondents.

Characteristic	N = 11[1]
access to training for new providers	
Can access training for new providers	6 (55%)
Cannot access training for new providers	5 (45%)

[1] n (%)

BCG Vaccination Uptake

BCG Vaccination Uptake (01/09/2021 - 30/06/2022)

Note: One commissioner did not provide a response, and has been excluded from the below table. The BCG vaccination uptake percentage was either 60%-79% (n=4), 80%+ (n=3), or unknown (n=3). Uptake challenges included:

- Funding challenges
- Clinic accessibility, and particularly travel distance
- Available appointment times
- Appointment invites and reminders
- Staffing challenges
- High DNA rates
- Language barriers
- Families struggle to attend appointments within the 28 day timeframe
- Parents want more time to consider vaccination
- Cross-border challenges where infants are offered appointments by two different services

Characteristic	N = 10[1]
BCG vaccination uptake	
60% - 79%	4 (40%)
80%+	3 (30%)
Unknown	3 (30%)

[1] n (%)

BCG Vaccination 28 Days (01/09/2021 - 30/06/2022)

The percentage of BCG vaccinations delivered within 28 days were <20% (n=2), 20% - 39% (n=3) and 40%-59% (n=5). Note: One commissioner did not provide a response, and has been excluded from the below table.

Characteristic	N = 10[1]
BCG vaccinations delivered within 28 days	
40% - 59%	5 (50%)
20% - 39%	3 (30%)
< 20%	2 (20%)

[1] n (%)

Case Studies

Seven commissioners (64%) outlined case study examples, which included:

-

Revising the national template to make it easier to complete
- Initiating an additional clinic in an alternative location to improve accessibility
- Phoning parents when they DNA during the appointment slot to discuss why they didn't attend and answer queries around vaccination
- Time spent training providers on correct completion of the monthly data returns
- Amending S4N when eligibility errors are noted
- Good working relationships between maternity and other providers to ensure contraindications (e.g. HIV) are shared with providers
- Selecting clinic locations based on heat mapping where families live
- Regional BCG pathway referrals document created and cascaded to all GP practices and health visitors so they are aware of the correct service to refer into if a child's eligibility was missed at birth or if they moved into the area
- Designated maternity staff who audit eligibility by getting NIPE practitioners to complete which country of origin as well as ticking correct box so coordinator can cross check if eligibility is correct
- Specialist community school aged immunisation service offer detailed conversations about the vaccination, and offer flexibility in clinic location and timings
- CHIS provider has developed an excellent system
- Some new pathways have been implemented to identify movers in and babies born out of county
- Reviewing the skill mix of staff delivering the service and bringing in staff from other services to improve the resilience of services
- Training session delivered to share good practice for completing data returns

There were not any commissioners that had completed an evaluation of their case studies.
