# Supplementary material for: A new neonatal BCG vaccination pathway in England: a mixed methods evaluation of its implementation
Source: BMC Public Health. 2024 Apr 26;24:1175. doi: 10.1186/s12889-024-18586-8 (PMC11046867; doi:10.1186/s12889-024-18586-8)
Supplement: Supplementary file 6 — Supplementary Material 6 [file 12889_2024_18586_MOESM6_ESM.pdf]

UK Health
Security
Agency

Evaluation Survey: Provider Data

Provider Overview

Region

Fifty four responses were received from BCG vaccination providers, covering all of the seven NHS regions. The most responses were received from the Midlands (n=14), followed by London (n=10) and the South West (n=10). The fewest responses were received from East of England (n=1).

Characteristic	N = 54[1]
respondent region	
Midlands	14 (26%)
London	10 (19%)
South West	10 (19%)
North West	8 (15%)
South East	7 (13%)

[1] n (%)

Characteristic	N = 54[1]
North East and Yorkshire	4 (7.4%)
East of England	1 (1.9%)

[1] n (%)

Local Authority

The BCG vaccination providers covered 93/153 local authorities in England. In 37 local authorities, there were two or more BCG vaccination providers.

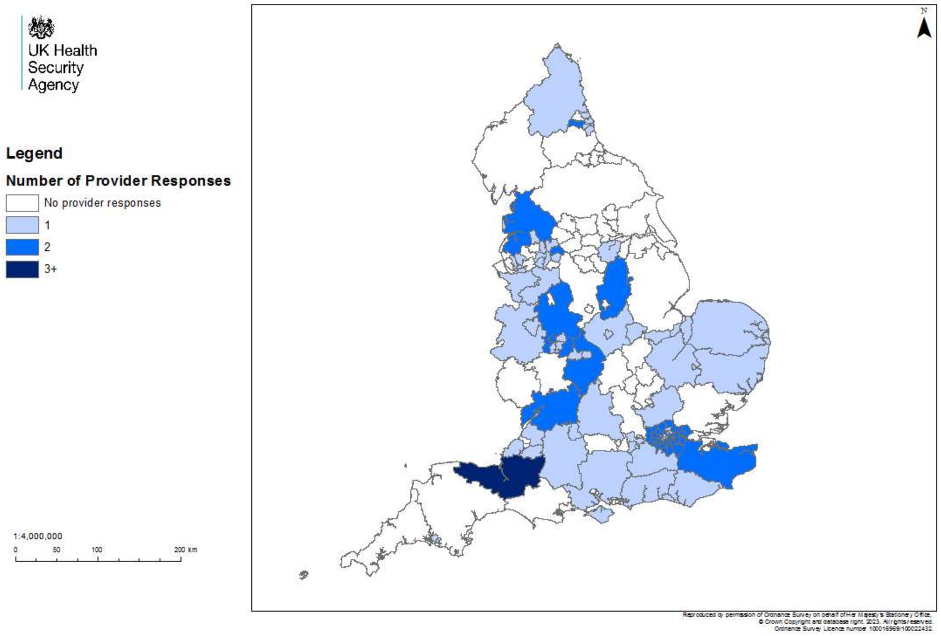

Provider Type

Responses were received from several different provider types, with the most responses from hospital trusts (44%), and the least responses from GP, TB services, and specialist BCG clinics (3.7% of total responses each).

Characteristic	N = 54[1]
provider type	
Hospital trust	24 (44%)
Community trust	9 (17%)
Maternity service	9 (17%)
Other	3 (5.6%)
Outpatient clinic	3 (5.6%)
GP	2 (3.7%)
Specialist BCG clinic	2 (3.7%)
TB service	2 (3.7%)

[1] n (%)

Provider Type by Region

There was variation in the most common provider type by region. Hospital trust was the most common provider type in the Midlands, North East and Yorkshire, North West and South West, whilst community trust was the most common provider type in East of England and London. In the South East, maternity service was the most common provider type.

Characteristic	**East of England**, N = 1[1]	**London**, N = 10[1]	**Midlands**, N = 14[1]	**North East and Yorkshire**, N = 4[1]	**North West**, N = 8[1]
provider type					

[1] n (%)

Characteristic	East of England, N = 1[1]	London, N = 10[1]	Midlands, N = 14[1]	North East and Yorkshire, N = 4[1]	North West, N = 8[1]
Hospital trust	0 (0%)	2 (20%)	8 (57%)	3 (75%)	4 (50%)
Community trust	1 (100%)	5 (50%)	1 (7.1%)	0 (0%)	1 (12%)
Maternity service	0 (0%)	0 (0%)	3 (21%)	0 (0%)	2 (25%)
Other	0 (0%)	2 (20%)	0 (0%)	0 (0%)	0 (0%)
Outpatient clinic	0 (0%)	0 (0%)	0 (0%)	1 (25%)	1 (12%)
GP	0 (0%)	1 (10%)	0 (0%)	0 (0%)	0 (0%)
Specialist BCG clinic	0 (0%)	0 (0%)	1 (7.1%)	0 (0%)	0 (0%)
TB service	0 (0%)	0 (0%)	1 (7.1%)	0 (0%)	0 (0%)

[1] n (%)

Provider Commissioned Prior the BCG Vaccination Change in 2021

Responses indicated that 52/54 (96%) current providers were also commissioned to deliver the BCG vaccination prior to the change implemented in September 2021.

Characteristic	N = 54[1]
provider commissioned	
provider previously commissioned (pre-September 2021)	52 (96%)
provider newly commissioned (post-September 2021)	2 (3.7%)

[1] n (%)

Geographical Coverage and Accessibility

BCG Vaccination Sites per Local Authority Covered

All responses (100%) indicated that there were between 1 and 9 BCG vaccination sites available per local authority covered.

Characteristic	N = 54[1]
number of sites available	
1 to 9	54 (100%)

[1] n (%)

Maximum Travel Distance to Access BCG Vaccination Clinics

The maximum distance that patients are required to travel to access BCG vaccination clinics ranged from 0 to 4 miles, to 20 miles or more, with 10 to 14 miles the most common response

(26%). 57% of providers reported a maximum travel distance of at least 10 miles.

Characteristic	N = 54[1]
maximum travel distance	
0 to 4 miles	10 (19%)
5 to 9 miles	13 (24%)
10 to 14 miles	14 (26%)
15 to 19 miles	4 (7.4%)
20 miles or more	13 (24%)

[1] n (%)

Frequency of Clinics

Clinic frequency ranged from less frequently than once a month (1.9%) to more frequently than once a week (31%). The most common clinic frequency was once a week (37%).

Characteristic	N = 54[1]
frequency of clinics	
More frequently than once a week	17 (31%)
Once a week	20 (37%)
Once a fortnight	12 (22%)
Once a month	4 (7.4%)
Less frequently than once a month	1 (1.9%)

[1] n (%)

Clinic Times

Daytime only clinics were the most common (76%), followed by a combination of daytime only and weekend clinics (11%).

Characteristic	N = 54[1]
clinic times	
Daytime only clinics	41 (76%)
Daytime only clinics;Weekend clinics	6 (11%)
Daytime only clinics;Evening clinics	2 (3.7%)
Weekend clinics	2 (3.7%)
Daytime only clinics;Evening clinics;Weekend clinics	1 (1.9%)
Evening clinics	1 (1.9%)
Home Visits;Daytime only clinics;Weekend clinics	1 (1.9%)

[1] n (%)

Accessibility Assessment

An assessment of the accessibility of clinic sites and appointment timings had been made by 30 providers (56%).

Where an accessibility assessment had been completed, several providers reported that the clinics were accessible and well attended, offering a choice of locations and appointment times for families. Some providers also noted that clinics were on regular public transport route, and one provider highlighted that families could request a home visit if there was a problem with clinic accessibility. Other providers reported that they planned to increase the number of available locations and the number of appointments offered.

Characteristic	N = 54[1]
accessibility assessment completed	
accessibility assessment completed	30 (56%)
accessibility assessment not completed	24 (44%)

[1] n (%)

Service Components

Process for Assessing Infants' BCG Eligibility Status

The process for assessing infants' BCG eligibility status is working effectively for 98% of providers.

Challenges identified related to assessing infants' BCG eligibility status included inappropriate referrals, eligibility not completed on S4N, delays in receiving results and staffing challenges. It was also noted that there can be challenges when infants have moved into an area covered by the provider from another area or from abroad.

Characteristic	N = 54[1]
eligibility process	
eligibility process working	53 (98%)
eligibility process not working	1 (1.9%)

[1] n (%)

Use of S4N

Use of S4N* was reported by 74% of providers.

*The new Newborn and Physical Examination (NIPE) IT system, SMaRT4NIPE (S4N) went live on 1 April 2019.

Characteristic	N = 54[1]
S4N	
S4N used	40 (74%)
S4N not used	14 (26%)

[1] n (%)

Effectiveness of the Referral Process to the BCG Immunisation Provider

The referral process to the BCG immunisation provider was considered to be effective by 85% of providers.

Challenges identified with the BCG referral process included out of area infants not being referred in a timely manner, challenges meeting the 28 day target for BCG vaccination due to referral delays, patients missing appointments, and eligible infants not being referred.

Characteristic	N = 54[1]
BCG referral	
Referral process effective	46 (85%)

[1] n (%)

Characteristic	N = 54[1]
Referral process not effective	8 (15%)

[1] n (%)

BCG Vaccination Appointment Booking

The BCG vaccination appointment is booked at referral by the majority of providers (74%), whilst 26% book the appointment when the SCID result has been received. Note: One provider did not provide a response, and has been excluded from the below table.

Characteristic	N = 53[1]
vaccination appointment booking	
At referral	39 (74%)
When the SCID result has been received	14 (26%)

[1] n (%)

Clinician Responsible for Assessing SCID Screening Outcome

Nurses (64%) were the clinician most commonly responsible for assessing the outcome of SCID screening for infants eligible for BCG vaccination, followed by other healthcare professionals (23%). Note: One provider did not provide a response, and has been excluded from the below table.

Characteristic	N = 53[1]
SCID assessment clinician	
Nurse	34 (64%)
Other healthcare professional	12 (23%)
Various clinicians	4 (7.5%)
Physician	2 (3.8%)
Unknown	1 (1.9%)

[1] n (%)

Effectiveness of current SCID Screening Outcome Process

The current SCID screening outcome process was considered to be effective by 87% of providers. Note: One provider did not provide a response, and has been excluded from the below table.

Challenges with the current SCID screening outcome process were predominantly around delays in receiving results and difficulties in obtaining SCID results for out of area infants, resulting in appointment cancellations and difficulties meeting the 28 day target for vaccination. Other responses noted that the current process is working well, or changes are being implemented to further improve the SCID screening outcome process.

Characteristic	N = 53[1]
effectiveness of SCID screening outcome process	
SCID screening outcome effective	46 (87%)

[1] n (%)

Characteristic	N = 53[1]
SCID screening outcome not effective	7 (13%)

[1] n (%)

Rescheduling BCG Vaccination Appointments due to Challenges Accessing SCID Results

38% of providers reported that they have had to reschedule at least one BCG vaccination appointment due to challenges accessing SCID results prior to the scheduled vaccination appointment. Note: One provider did not provide a response, and has been excluded from the below table.

The providers noted that BCG vaccination appointments were most often rescheduled due to delays in obtaining the SCID result, particularly when repeat sampling is required.

Characteristic	N = 53[1]
vaccine appointment rescheduled	
At least one vaccine appointment rescheduled	20 (38%)
No vaccine appointments rescheduled	33 (62%)

[1] n (%)

Method of Finding out ‘SCID Suspected’ Result

The most common way for providers to find out the SCID suspected result was from the immunology service (31%) followed by from CHIS (16%). Seven providers (14%) noted that

they had not yet had a SCID suspected result and were unsure how they would find out about the result. There were also five providers (10%) that were not part of the SCID pilot, and would therefore not receive the SCID result.

Note: Five providers did not provide a response, and have been excluded from the below table.

Note: Responses to this question have been categorised during data cleaning due to significant use of free text. There were several instances where providers specified that a SCID result had not yet been received, but outlined which method would be used (e.g. ‘have not yet received a SCID suspected result, but would be notified by the immunology service’). Where a provider has noted the method of finding out the SCID result, this has been prioritised over noting that the provider has not yet received a SCID result.

**Characteristic**	**N = 49**[1]
method of obtaining SCID screening outcome result	
From the immunology service	15 (31%)
CHIS	8 (16%)
Unsure, have not had a SCID suspected result	7 (14%)
N/A - area not part of SCID pilot	5 (10%)
From a clinician (not GP)	3 (6.1%)
From a GP	3 (6.1%)
From a GP;From the immunology service	3 (6.1%)
Result is not received by provider	2 (4.1%)
From a clinician	1 (2.0%)

[1] n (%)

Characteristic	N = 49[1]
From a GP;From the immunology service;From the parents	1 (2.0%)
From a GP;From the parents	1 (2.0%)

[1] n (%)

BCG Vaccination Appointment Delay due to Method of Finding Out SCID Screening Result

Excluding responses where the method of finding out the SCID result was ‘no response’, ‘N/A - area not part of SCID pilot’, or ‘Unsure, have not had a SCID suspected result’, 41% of providers reported that BCG vaccination appointments had been delayed due to the method of finding out the SCID screening results.

Characteristic	N = 37[1]
BCG vaccination appointment delayed due to SCID screening result method	
BCG vaccination appointment delayed	15 (41%)
BCG vaccination appointment not delayed	22 (59%)

[1] n (%)

Call and Recall

BCG Vaccination Invite Format

Letter was the most common form of vaccination invite (43%) followed by phone (15%). There were providers that used multiple appointment invitation methods, including text and letter (13%) and phone and letter (13%). There was one provider that used all four vaccination invite methods (at discharge from postnatal ward, phone, text and letter).

Characteristic	N = 54[1]
format of vaccination invite	
Letter	23 (43%)
Phone	8 (15%)
Phone;Letter	7 (13%)
Text;Letter	7 (13%)
Phone;Text;Letter	3 (5.6%)
At discharge from postnatal ward	2 (3.7%)
Phone;Text	2 (3.7%)
At discharge from postnatal ward;Letter	1 (1.9%)
At discharge from postnatal ward;Phone;Text;Letter	1 (1.9%)

[1] n (%)

Number of BCG Vaccination

Invite Reminders

The providers reported sending between 0 and 3+ reminders, with 1 reminder the most common (33%).

Characteristic	N = 54[1]
number of reminder invitations	
0	12 (22%)
1	18 (33%)
2	16 (30%)
3+	8 (15%)

[1] n (%)

DNA Rates

BCG vaccination DNA rates ranged from <20% (48% of providers) to 40%-59% (6% of providers).

Characteristic	N = 54[1]
DNA rates	
<20%	26 (48%)
20% - 39%	25 (46%)
40% - 59%	3 (5.6%)

[1] n (%)

DNA Rates and Invite

Reminders

The lowest DNA rates were observed where 3+ reminder invitations were sent, with a <20% DNA rate achieved by 62% of providers sending 3+ reminder invitations. Where 0 reminders were sent, a <20% DNA rate was achieved by 42% of providers. The highest DNA rate (40% - 59%) was reported by providers who sent out 1 or 2 reminder invitations.

**Characteristic**	**<20%**, N = 26[1]	**20% - 39%**, N = 25[1]	**40% - 59%**, N = 3[1]
number of reminder invitations			
0	5 (42%)	7 (58%)	0 (0%)
1	8 (44%)	8 (44%)	2 (11%)
2	8 (50%)	7 (44%)	1 (6.2%)
3+	5 (62%)	3 (38%)	0 (0%)

[1] n (%)

Information Tailored to the Population Served

UKHSA BCG Vaccination Leaflet

The UKHSA BCG vaccination leaflet is given to parents or guardians by 50 providers (93%) (yes = 44, sometimes = 6)

Characteristic	N = 54[1]
UKHSA leaflet given to parents or guardians	
Yes	44 (81%)
Sometimes	6 (11%)
No	4 (7.4%)

[1] n (%)

Local BCG Vaccination Leaflet

A local BCG vaccination leaflet is given to parents and guardians by 27 providers (50%) (yes = 23, sometimes = 4).

Characteristic	N = 54[1]
local leaflet given to parents or guardians	
No	27 (50%)
Yes	23 (43%)
Sometimes	4 (7.4%)

[1] n (%)

Where the local leaflet was given (n=27), it was most often given at the vaccine appointment (48%), followed by with the vaccine invite (26%).

Characteristic	N = 27[1]
Timing of giving the local leaflet given to parents or guardians	

[1] n (%)

Characteristic	N = 27[1]
At the vaccine appointment	13 (48%)
With vaccine invite	7 (26%)
Other	5 (19%)
When booking vaccine appointment	2 (7.4%)

[1] n (%)

Where the local leaflet was given (n=27), 17 providers (63%) reported that it was available in other languages. The available languages varied between providers, with some providing the leaflet in only one other language, whilst others were able to translate the leaflet according to patient requirements.

Characteristic	N = 27[1]
local leaflet provided in other languages	
Yes	17 (63%)
No	8 (30%)
no response	2 (7.4%)

[1] n (%)

Additional Information Provided

Additional information is provided to parents and guardians by 40 providers (74%) (yes = 31, sometimes = 9).

Characteristic	N = 54[1]
additional information provided	

[1] n (%)

Characteristic	N = 54[1]
Yes	31 (57%)
No	14 (26%)
Sometimes	9 (17%)

[1] n (%)

Where details about additional information was provided (n=31), clinical advice (58%) was the most common, followed by a combination of clinical advice and website links (23%).

Characteristic	N = 31[1]
type of additional information provided	
Clinical advice	18 (58%)
Clinical advice;Website links	7 (23%)
Website links	4 (13%)
Other	2 (6.5%)

[1] n (%)

Where details about additional information was provided (n=31), information was most often given to parents or guardians at the vaccine appointment (65%), followed by with the vaccine invite (16%).

Characteristic	N = 31[1]
Timing of providing additional information	
At the vaccine appointment	20 (65%)
With vaccine invite	5 (16%)

[1] n (%)

Characteristic	N = 31[1]
Other	3 (9.7%)
When booking vaccine appointment	3 (9.7%)

[1] n (%)

Where details about additional information was provided (n=31), 16 providers (48%) reported that the information was available in other languages.

Characteristic	N = 31[1]
additional information provided in other languages	
Additional information provided in English only	16 (52%)
Additional information provided in other languages	14 (45%)
no response	1 (3.2%)

[1] n (%)

Further Comments

Several providers noted the challenges of delivering the new BCG vaccination programme. The challenges highlighted included additional workload, high DNA rate and cancellation of appointments at short notice, decrease in vaccination uptake, delay in receiving SCID results, and difficulty meeting the 28 day target for BCG vaccination.
